# Review of NiS-Based Electrode Nanomaterials for Supercapacitors

**DOI:** 10.3390/nano13060979

**Published:** 2023-03-08

**Authors:** Yuhao Guan, Kexie Hu, Nan Su, Gaohe Zhang, Yujia Han, Minrong An

**Affiliations:** 1 College of New Energy, Xi’an Shiyou University, Xi’an 710065, China; 2 College of Electrical Engineering, Xi’an Jiaotong University, Xi’an 710049, China; 3 Engineering Science and Technology College of Equipment Engineering, Shanxi Vocational University of Engineering and Technology, Taiyuan 030619, China; 4 Shanxi Research Institute for Clean Energy, Tsinghua University, Taiyuan 030032, China

**Keywords:** NiS-based electrode materials, supercapacitors, electrochemical properties

## Abstract

As a new type of energy storage device, supercapacitors have the advantages of high-power densities, high safety factors, and low maintenance costs, so they have attracted widespread attention among researchers. However, a major problem with supercapacitors is that their energy densities are not high enough, which limits their application. Therefore, it is crucial to expand the application scenarios of supercapacitors to increase their energy density as much as possible without diminishing their advantages. The classification and working principles of supercapacitors are introduced in this paper. The electrochemical properties of pure NiS materials, NiS composites with carbon materials, NiS composites with sulfide materials, and NiS composites with transition metal oxides for supercapacitors are summarized. This paper may assist in the design of new electrode materials for NiS-based supercapacitors.

## 1. Introduction

With the aggravation of the global energy crisis and environmental pollution, global energy consumption is accelerating at an astonishing rate [1,2,3,4,5]. Energy shortages and environmental pollution have become the two major challenges facing current development [6,7,8,9]. Due to the popularity of portable electronic equipment and the rapid development of new powered vehicles, human society urgently needs to develop clean, sustainable, and renewable energy [10,11,12,13,14,15,16]. Against this background, the supercapacitor—a new type of green energy storage device—has been developed. High power, a long life, and high reliability are the advantages of double-layer supercapacitors [17,18,19,20], which have been widely used in multifunctional electronic products, power systems, military components, and other high-peak-power fields [21,22,23,24,25].

According to their energy storage mechanism, supercapacitors [26,27,28,29,30] can be divided into two categories: the energy storage mechanism of capacitors using activated carbon with a high specific surface area is based on the double-layer capacitance generated by charge separation at the carbon electrode–electrolyte interface [31,32], and capacitors using RuO_2_ and other noble-metal oxides as electrodes use adsorbed capacitances generated by redox reactions on the surface and the bulk phase of oxide electrodes, which is called Faraday quasicapacitance [33,34,35]. Because the generation mechanism of Faraday capacitance is similar to that of a battery reaction, its capacitance is several times that of double-layer capacitance when the electrode area is the same. However, the power characteristic of double-layer capacitors is better than that of Faraday capacitors. It is generally believed that supercapacitors are a new type of energy storage component between traditional capacitors and batteries, with some characteristics of both [36,37,38,39]. They are widely used in the fields of high-power pulse power supply and electric vehicle drive power supply [40,41,42,43,44].

**Figure 1:**
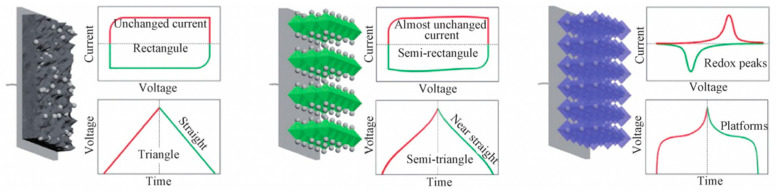
Comparison of the energy storage mechanisms of different materials [45].

Supercapacitors can be divided into three categories based on the differences [45] in their energy storage mechanisms (see Figure 1). The first type is the double-layer supercapacitor. This capacitor’s capacity is generated by the accumulation of electrostatic charge at the electrode–electrolyte interface. This is a purely physical process that does not involve redox reactions. The main electrode materials of double-layer capacitors are carbon materials, including activated carbon, carbon nanotubes, and graphene. These carbon materials have a low cost, high conductivity, high magnification, and can easily be used to form porous structures, but they have a low capacity. The second type is the pseudocapacitor, whose electrode materials mainly include metal oxides, metal hydroxides, metal sulfides, and conductive polymers. These materials store and release energy by undergoing a rapid, reversible redox reaction at the electrode surface. Both of these mechanisms react on the surface of the electrode. If the electrode’s design is reasonable, the third type of supercapacitor—the hybrid supercapacitor—can be formed by using double-layer capacitance and pseudocapacitance at the same time. Hybrid supercapacitors are mainly composed of a Faraday battery positive electrode and a double-layer negative electrode, so they are also called asymmetric supercapacitors.

As a symbol of the performance of supercapacitors, the electrode materials have spurred debate among scholars both at home and abroad. With the development of nanotechnology, various kinds of electrode materials with different structures have been designed and studied [46,47,48,49,50,51,52]. The electrode materials of supercapacitors include sulfides [53,54,55], carbon materials [56,57,58], oxides [59,60,61], nitrogen materials [62,63], conductive polymers [64], etc. In recent years, transition metal oxides (TMOs) have become some of the most promising materials for pseudocapacitor electrodes due to their excellent chemical stability, impurity sensitivity, environmental compatibility, and resource advantages [65]. The most commonly used TMOs are ruthenium dioxide and manganese dioxide. Because the charge storage mechanism of pseudocapacitors is closer to that of batteries than to that of capacitors, the specific capacitance that they can provide is one order of magnitude higher than that of carbon-based EDLCs. For example, the specific capacitance of RuO_2_ pseudocapacitors can reach 768 F g^−1^, or 150–250 F cm^−2^.

Accordingly, the difference in the mechanism also gives the transition metal oxide electrode different performance-limiting factors than EDLCs. On the one hand, due to the speed limitations of electron conduction and ion diffusion, the atoms at a certain depth below the surface of the electrode materials participate in the redox reaction very slowly, or sometimes cannot participate in the reaction at all, which severely limits the ratio performance and specific capacity of the materials. On the other hand, due to the irreversible damage to the structure caused by the repeated phase transition experienced by the electrode materials in the process of charge and discharge, the cycle life of the electrode cannot be compared with that of an EDLC, which relies on electrostatic force to store the charge. These two factors are the bottlenecks that limit the performance optimization and application of pseudocapacitors, and these problems have puzzled researchers for a long time. Compared with monometal oxides, binary and ternary transition metal oxides have the advantages of richer redox reactions, higher electrochemical activity, and stronger electrical conductivity. Therefore, the preparation and electrochemical application of binary and ternary transition metal oxides have attracted extensive attention in recent years.

At present, there are three strategies used to improve the electrochemical performance of transition metal oxides [66]: (1) obtain more abundant redox states and good electronic conductivity by synthesizing polymetallic oxides; (2) construct a reasonable hierarchical structure to shorten the ionic diffusion distance, so as to provide more reactive sites; or (3) combine transition metal oxides with highly conductive materials to form nanocomposites. Manickam Minakshi et al. [67] studied the effect of Zn doping on the formation of surface defects on the structure of nickel molybdate (NiMoO_4_) with different zinc contents, and they prepared one-dimensional electrodes and catalysts for electrochemical energy storage and ethanol oxidation, respectively, which were synthesized from simple wet-chemical-doped zinc nickel molybdenum acid (Ni_1−x_Zn_x_MoO_4_, where x = 0.1, 0.2, 0.4, and 0.6) nanorods. Compared with the original NiMoO_4_, the Zn-doped NiMoO_4_ with optimal Zn content (the optimal Zn content was ~25%) was tested as the electrode of an asymmetric supercapacitor. At a high power density of 384 W kg^−1^, the specific capacitance was enhanced by 122 F g^−1^, and the specific energy density was 43 Wh kg^−1^. Through the formation of excess oxygen vacancies, the dopants played an important role in enhancing the charge transfer between the electrolyte surface and the OH^-^ ions, which made the Zn doping result in good conductivity.

In a different study, Manickam Minakshi et al. [68] used Zn-doped nickel molybdate (NiMoO_4_) (ZNM) as the core crystal structure and AWO_4_ (a = Co or Mg) as the shell surface. Based on density functional theory, the interface model of the Zn-doped NiMoO_4_@AWO_4_ (ZNM@AW) core–shell structure was simulated. The morphology and electrochemical properties of the layered ZNM@AW core–shell semiconductor nanocomposites were characterized by using a simple and green two-step hydrothermal method after being grown on a conductive nickel foam substrate. The performance of the core–shell structure was significantly affected by the inherent properties of the selected metal oxides, and it exhibited better chemical and electrochemical properties than the single-component systems in supercapacitors. Further recent studies of asymmetric devices doped with NiMoO_4_@CoWO_4_ (ZNM @ the CW) and activated carbon showed excellent electrochemical performance. For a current density of 2 mA cm^−2^, the surface capacitance was 0.892 F cm^−2^. After 1000 cycles of charge and discharge, the initial capacitance maintained 96% of its cycle life.

**Figure 2:**
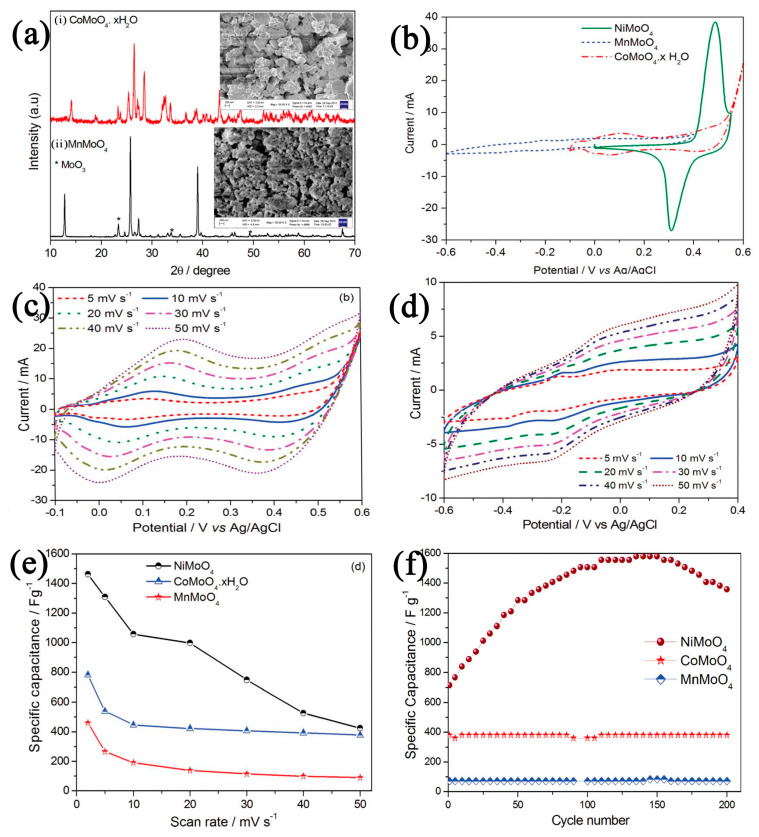
(**a**) XRD pattern and FE-SEM image (inset) of as-prepared (i) CoMoO_4_·xH_2_O and (ii) MnMoO_4_ (The asterisk refers to the diffraction peak position of MoO_3_ phase); (**b**) CV curves of α-NiMoO_4_, MnMoO_4_, and CoMoO_4_·xH_2_O at a scan rate of 5 mV s^−1^; (**c**) CoMoO_4_·xH_2_O and (**d**) MnMoO_4_ at different scan rates from 5 to 50 mV s^−1^; (**e**) the SC as a function of the scan rate; (**f**) variation in the SC with cycle number at 12 A g^−1^ for α-NiMoO_4_, MnMoO_4_, and CoMoO_4_ [69].

In another study, Manickam Minakshi et al. [69] prepared nanosized nickel molybdate (α-NiMoO_4_) via solution combustion synthesis (SCS). Compared with MnMoO_4_ and NiMoO_4_·xH_2_O, α-NiMoO_4_ showed a higher electrochemical capacitance performance due to the rapid reversible redox reaction of nickel. Although the cyclic stability of α-NiMoO_4_ was inferior to that of MnMoO_4_ and CoMoO_4_·xH_2_O, α-NiMoO_4_ was far superior in terms of electrochemical behavior. Due to the pseudocapacitance property of the nano-α-NiMoO_4_ material, it has a higher specific capacitance (1517 F g^−1^) and energy density (52.7 Wh Kg^−1^) at a current density of 1.2 A g^−1^ (see Figure 2). Wei et al. [70] anchored fluffy Co_3_O_4_ nanowires on the surface of Co_3_O_4_ nanosheets. The results showed that the fractional structure composed of nanosheets and nanowires increased the specific surface area of the material, shortened the ion diffusion path, and enabled the mass-specific capacitance of the material to reach 2053.1 F g^−1^ at a current density of 1 A g^−1^. Wang et al. [71] used a hydrothermal method to grow NiMoO_4_ nanomaterials on the surface of nickel foam. The morphology of the materials was regulated by changing the reaction time, and NiMoO_4_ nanomaterials with a high crystallinity and large specific surface area were prepared via a reaction at 150 °C for 6 h. At the current density of 5 mA cm^−2^, the area-specific capacitance of NiMoO_4_ could reach 3.48 F cm^−2^.

The transition metal hydroxide is represented by Ni(OH)_2_. Although Ni(OH)_2_ has a relatively high theoretical specific capacity, the specific capacity of the material will be attenuated significantly during the process of charging and discharging. Therefore, bimetallic hydroxides are mainly used to replace single metallic hydroxides to improve the structural stability of the materials. Nath et al. [54] prepared nickel sulfide–nickel hydroxide (NSH) composite layers on nickel foam electrodes for asymmetric supercapacitors via successive ion layer adsorption and reaction (SILAR). Composite-coated nickel foam (NF) substrates provided a specific capacity of 108 C g^−1^ in a 3 A g^−1^ three-electrode system. At a potential window of 0.65 V, the electrode also showed good stability at a high current density of 50 A g^−1^. The coexistence of nickel sulfide and nickel hydroxide in the composite resulted in excellent electrochemical properties. An asymmetric supercapacitor (ASC) device (NSH//KOH//MAC) was constructed with NSH as the positive electrode and modified activated carbon (MAC) as the negative electrode. The maximum energy and power density of the ASC were 51 Wh kg^−1^ and 18 kW kg^−1^, respectively.

To date, the serious defect in research regarding electrode materials is that the energy density is not high enough to meet the demand [72,73]. In recent years, transition metal sulfides have received extensive attention due to their superior electrical conductivity and electrochemical activity compared to transition metal oxides/hydroxides. Typically, transition elements from groups IV to VIIB combine with group VIA elements such as S, Se, and Te to form a binary, stable, layered crystal structure. The general formula for these layered transition metal sulfide compounds is MX_2_, where M is IVB (Ti, Zr, Hf), VB (V, Nb, Ta), VIB (Mo, W), or VIIB (Tc, Re), while X is the sulfur atom of the VIA group (S, Se, Te). Metal sulfides show good electrochemical properties, mainly due to their high electrical conductivity and their excellent mechanical and thermal stability [74]. However, the performance of electrochemical energy storage devices depends largely on the crystalline phase; the size, structure, and morphological characteristics of the active material; and the composition and design of the electrode. Fortunately, among the many transition metals, nickel exhibits rich valence states, so elemental nickel and sulfur can form compounds with a variety of crystal structures (including NiS, NiS_2_, Ni_2_S_3_, Ni_3_S_4_, etc.). Among them, NiS stands out from all kinds of nickel sulfides due to its rich chemical composition, high specific capacity, excellent electrochemical activity, and environmental friendliness [75,76]. The mechanism of lithium removal by nickel sulfide is different from that of other layered sulfides. In general, nickel sulfide is reduced to elemental Ni and is accompanied by the formation of Li_2_S. The process of removing lithium produces a corresponding nickel sulfide and releases Li^+^. The NiS electrode has good electrical conductivity and a high specific capacity (590 mAh g^−1^) [77,78,79,80]. Therefore, NiS-based materials [81,82] can effectively solve the above problems and may be potential candidates for supercapacitors. In view of the current development status of nickel sulfide electrode materials, in this paper, we review the research status of nickel sulfide materials, nickel sulfide composites with carbon materials, and nickel sulfide composites with oxide materials as the electrode materials of supercapacitors. In addition, future research directions for nickel sulfide materials used as electrode materials for supercapacitors are proposed.

## 2. Pure NiS Nanomaterials

Generally, NiS exhibits two possible phases: α-NiS (hexagonal, P63/mmc) and β-NiS (rhombohedral, R3m). For the two structures of NiS, various morphological attempts have been applied to modify their electrochemical performance. Moreover, the synthesis methods have led to nanolayers, nanospheres, and other shapes. These different shapes enhance the ion transport to different extents. The details of these electrodes can be summarized as follows.

### 2.1. NiS Nanolayered Structure

**Figure 3:**
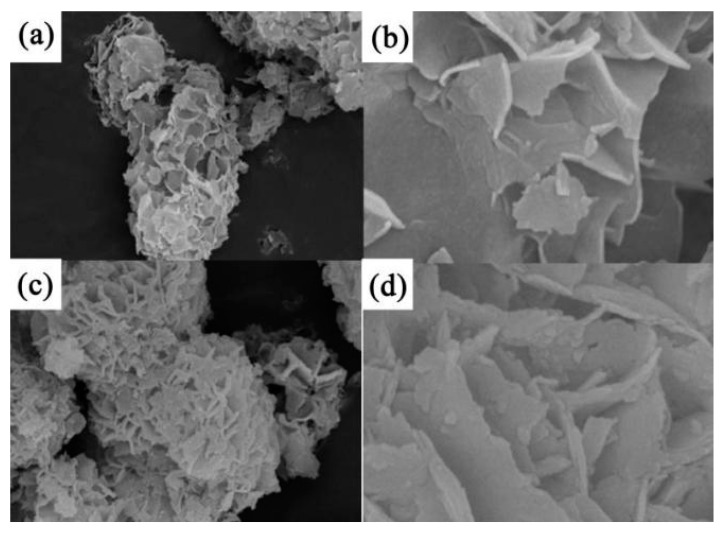
SEM images of (**a**,**b**) precursors and (**c**,**d**) NiS [83].

Guan et al. [83] synthesized hierarchical NiS microflowers via vulcanization by using Ni(OH)_2_ as a precursor. A SEM analysis showed that the NiS microflowers were composed of layered nanoplates whose surfaces were much rougher than the original template after vulcanization (see Figure 3).

**Figure 4:**
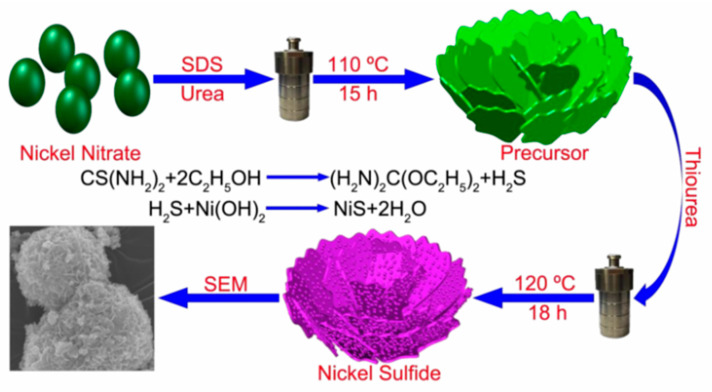
Formation process of NiS microflowers [84].

Electrochemical tests showed that the NiS had a high specific capacitance (i.e., when the current density was 1 A g^−1^, the specific capacitance was 1122.7 F g^−1^) and excellent electrochemical stability (i.e., after 1000 charging and discharging cycles, it retained 97.8% of its specific capacitance). In addition, an asymmetric supercapacitor with NiS as the positive electrode and activated carbon as the negative electrode had a high energy density under a 1.8 V operating voltage window. Parveen et al. [84] synthesized a hierarchical framework of mesoporous nickel sulfide using a simple solvothermal method and characterized it using a series of spectral and microscopic techniques. The formation process of NiS microflowers and the experimental synthesis process are shown in Figure 4. Symmetrical supercapacitors assembled from layered NiS showed high capacitance of 11.15 F g^−1^, an energy density of 0.991 Wh kg^−1^ when the power density was 132 W kg^−1^, and good cycling stability.

**Figure 5:**
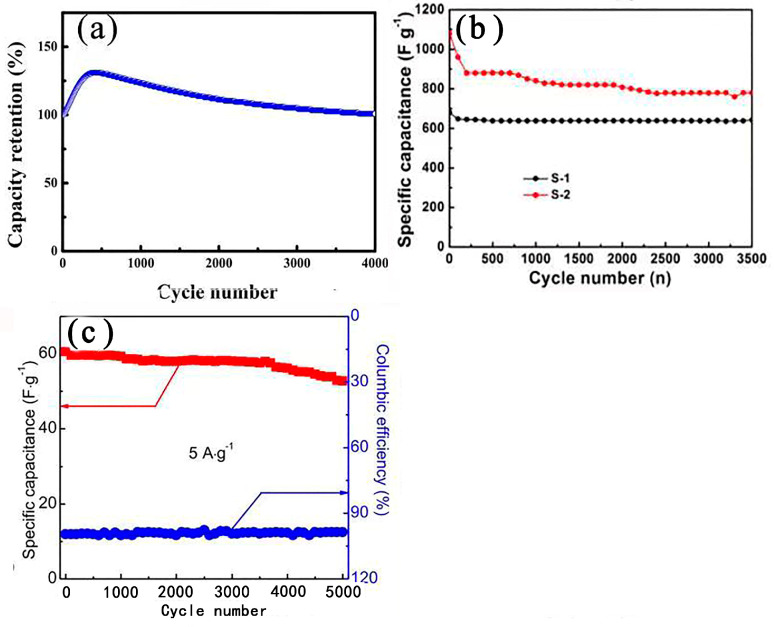
(**a**) Cycle stability [85]. (**b**) Rate performances of S-1 and S-2 [89]. (**c**) Cycle performance and Coulombic efficiency [92].

Li et al. [85] synthesized porous NiS hexagonal nanoplates (NiS HNPs) via the anion exchange method as electrode materials for supercapacitors. The excellent electrochemical performance of NiS HNPs has been shown to be due to their unique layered porous structure. The specific capacitance of porous NiS hexagonal nanoplates was 1897 F g^−1^ when the current density was 1 A g^−1^. NiS HNPs//activated carbon (AC) asymmetric supercapacitors (ASCs) had a long cycle life (i.e., capacity maintained at around 100% after 4000 cycles at a current density of 3 A g^−1^) (see Figure 5a). Yu et al. [86] used nickel hydroxide nanosheets as precursors to prepare interconnected staggered nickel sulfide nanosheets grown on nickel foam via an ultrasound-assisted soaking method. A SEM morphological analysis showed that the composition of NiS comprised hollow spherical nanoparticles. The nanosheet had a unique structure as an electrode material for supercapacitors and exhibited excellent electrochemical properties, including a high specific capacitance (2.64 F cm^−2^) and remarkable cyclic stability (90% after 2000 cycles). At the same time, an asymmetric supercapacitor was prepared by using NiS-NF as a positive electrode material and activated carbon as a negative electrode material. This device had good long-term electrochemical stability and a high energy density. Kang et al. [87] alternately dipped substrates deposited with TiO_2_ nanoparticles (for only 6 min per cycle) into nickel acetate and sodium sulfide solutions at room temperature. The amount of NiS deposited could be directly controlled by the number of deposition cycles. The maximum specific capacitance obtained was 1044 F g^−1^. The aim was to increase the surface area through the nanostructure of the electrode, taking full advantage of the superior electrochemical properties of nickel sulfide as the electrode material for the supercapacitors. In addition, the voltammetric response retention under high-speed scanning was improved by adding nanostructures (1.5 times the electric maximum of flat films). Yan et al. [88] synthesized NiS nanosheets on nickel foam via a hydrothermal method. The nickel foam was covered by dense NiS nanosheets. These micrometer-scale nanosheets were grown on the substrate to form a three-dimensional frame structure. The hydrothermal precursors on the nickel foam were characterized using high-power scanning electron microscopy. The device had an ultrahigh capacitance of 2587 F g^−1^ at a scan rate of 0.2 A g^−1^ (corresponding to a long discharge time of 5563 s). It had an excellent cyclic stability of 95.8% after 4000 cycles. The layered floral nickel sulfide provides more reaction sites for the charge–discharge process. The number of synthesized nanoplates can be adjusted by varying the amount of reactant added; the larger the number of nanoplates, the higher the specific capacitance of the sample, and the better the cyclic stability. After 3000 cycles, the specific capacitance remained at 778.8 F g^−1^ when the current density was 4 A g^−1^ [89] (see Figure 5b). Moreover, the hollow structure also inspired researchers to improve the performance. In another study, each NiS monolayer hollow sphere (MHSA) [90] was composed of 5–10 nm nanoparticles with excellent electrochemical properties, such as a fast ion/electron transport path and the large contact area between the electrolyte and the active material. The electrochemical test results showed a high capacity (i.e., 68.5 mAh g^−1^ at a current density of A g^−1^) and a good cycle life (65 mAh g^−1^ after 3000 cycles at 2 A g^−1^). By using α-Fe_2_O_3_ templates of different forms, a bishell hollow nickel sulfide composed of crossed ultrathin NiS nanosheets [91] with a controllable shape (i.e., cube, ellipsoid, and capsule) and uniform size was synthesized. Due to its high specific surface area (100.2 m^2^ g^−1^), low equivalent series resistance (0.8 Ω), and ionic diffusion resistance, the capsule structure had a maximum specific capacitance of 1159 F g^−1^ at a current density of 2 A g^−1^. Further research showed that the asymmetric capacitor NiS (capsule) || RGO @ Fe_3_O_4_ had a high energy density and good cycle stability.

### 2.2. NiS Nanosphere Structure

In a study by Tran et al., the structure of synthesized hierarchical NiS with nanoflowers using Ni(OH)_2_ microflowers [92] as precursors via a surface sacrifice template method under different curing times gradually changed from microspheres to nanospheres with the prolongation of the vulcanization time. Electrochemical tests showed that NiS-18 had the highest specific capacitance and good cyclic stability. An asymmetric supercapacitor (SC) consisting of NiS-18 as the positive electrode and activated carbon (AC) as the negative electrode (NiS//AC) achieved a high potential of 1.6 V. The NiS//AC SC exhibited an excellent cycle life (87.3% Cm retained after 5000 GCD cycles at current density of 5 A·g^−1^) and close to a 100% Coulomb efficiency (see Figure 5c). 

**Figure 6:**
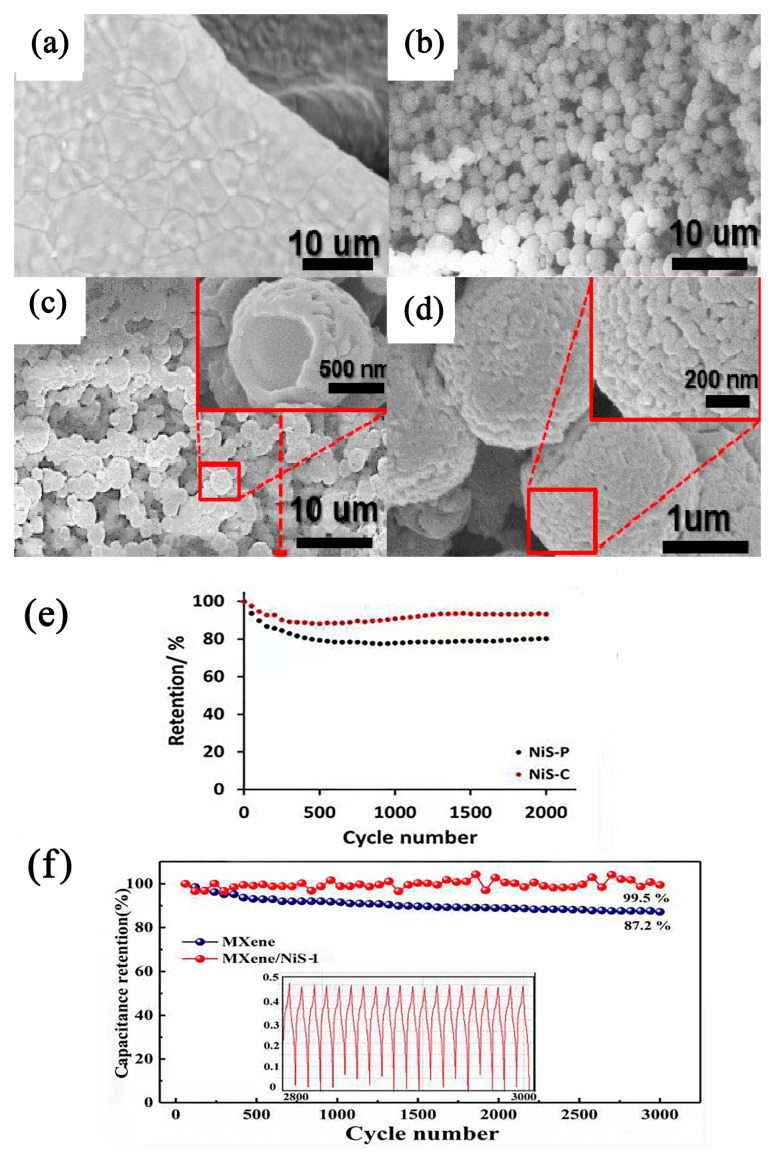
SEM images of (**a**) the bare NF and (**b**–**d**) NiS@NF at low and high magnifications [93]. (**e**) Cycles for NiS-P and NiS-C [94]. (**f**) Cycle performance of MXene and MXene-NiS-1 electrodes. The inset shows the last 20 cycles of an MXene/NiS-1 electrode [96].

Tran et al. [93] synthesized hollow NiS spheres directly on the surface of Ni foam (NiS@NF) at room temperature via electrodeposition. The SEM results showed the formation process (see Figure 6). At a current density of 2.35 A g^−1^, the NiS@NF nanocomposite had a very high specific capacitance (1553 F g^−1^). The NiS@NF nanocomposites exhibited excellent cycle stability (i.e., specific capacitance retention of 95.7% after 2000 cycles).

In a study by Harish et al., the hexagonal nanoplate of self-assembled hierarchical NiS microspheres [94] prepared using trimethylamine (TEA)-assisted hydrothermal methods reached 606 C g^−1^ at 0.5 A g^−1^. TEA played the role of a capping agent that can enhance the formation of nanoplates in the formation of the hierarchical NiS hexagonal nanoplate. The effective surface area of the electrolyte was increased by a morphological modification, which solved the problem of the incomplete utilization of the active substance at a high current density, thereby improving the rate performance of the electrode. Due to its porosity, the optimized sample retained 93% of its capacity after 2000 consecutive charge–discharge cycles. Furthermore, an asymmetric supercapacitor with NiS-C as the positive electrode and activated carbon as the negative electrode had a high energy density in the operating voltage window of 1.5 V (see Figure 6e). Nandhini et al. [95] synthesized nickel sulfide nanostructures (M, H, and MH) via microwave, hydrothermal, and microwave combined with hydrothermal methods. The X-ray diffraction (XRD) results showed that the Ni_9_S_8_ in M was in the quadrature phase, and the Ni_9_S_8_ of H and MH was in a hexagonal NiS structure. The SEM morphology of M, H, and MH were nanosheet, spherical, and lamellar structures, respectively. Electrochemical studies showed that the MH electrode had better performance than the other two electrodes and had a very high cycle life. Due to its special multilayered structure, the MH electrode had a higher specific capacitance, lower charge transfer resistance, and better electrochemical stability.

### 2.3. Other Nanoshapes of NiS

**Figure 7:**
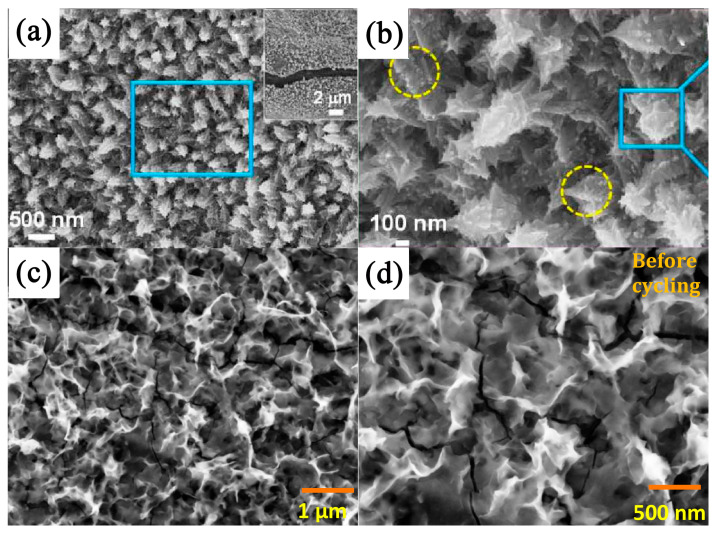
(**a**,**b**) When the growth time was further extended to 5 h, the nanobranches were densely oriented on the NiS nanopillars (indicated by the yellow circles), and the adhesion of the nanobranches was observed to be poor due to the overcoating of the material and the surface cracks [98]. (**c**,**d**) FE-SEM images of the NiS thin film surface at magnifications of 10,000× and 25,000×, respectively [99].

Liu et al. [96] prepared flower-like three-dimensional NiS on MXene (Ti_3_C_2_T_x_) via a one-step hydrothermal method. The nanosheets connected in the composite formed a large void network structure that facilitated the transfer of ions and charges. The MXene/NiS composites had a specific capacitance of 746 F g^−1^ at 1 A g^−1^—3.5 times that of MXene—and a capacitance retention rate of 99.49% after 3000 cycles at a current density of 5 A g^−1^ (see Figure 6f). Kumar et al. [97] used a continuous ion layer adsorption and reaction method on a nickel foam substrate. In the assembled solid-state asymmetric supercapacitors, the self-assembled NS-SnS (NTS) heterogeneous electrode materials exhibited a significant high specific capacitance value of 1653 F g^−1^ when the current density was 1 A g^−1^ and a significant high energy density of 83 Wh kg^−1^ when the power density was 117 W kg^−1^. They also had an excellent rate capability and cycle stability. Raju et al. [98] designed and grew three-dimensional porous nickel sulfide nanotrees with reliable adhesion on NiS NTs/Ni foam using wet chemistry (see Figure 7a,b). When the current density was 4 mA cm^−2^, the maximum area capacity and specific capacity of the forested NiS NTs/Ni foam were 752.71 μAh cm^−2^ and 342.1 mAh g^−1^, respectively, and the circulating stability was 89.4%. Due to its good hierarchical structure with more surface-active sites, it allowed for the rapid diffusion of electrolyte ions and minimized the electron transport limitations. The hybrid supercapacitor, using multilayer NiS NTs/Ni foam as a cathode and an active-carbon-based anode, had a 1.6 V potential window and high power density.

Patil et al. [99] prepared highly porous nanoflame nickel sulfide films on a flexible stainless steel substrate via chemical bath deposition (see Figure 7c,d). The nanoflame NiS films had a good electrochemical performance in a three-electrode system, and the maximum specific capacitance was 750.6 F g^−1^ at a scanning rate of 5 mV s^−1^. On this basis, portable symmetric flexible solid-state supercapacitors (FSS-SCs) and electrochemical supercapacitors (SCs) were prepared. The results of the comparison between the symmetrical FSS-SCs formed via the nanoflame NiS and the electrochemical SCs fully reflected the excellent electrochemical performance of the former.

The above studies show that NiS has a rich chemical composition, high specific capacity, excellent electrochemical activity and environmental friendliness, and exhibits a large storage capacity, high energy density, high power density, and good cyclic stability when used as an electrode material for supercapacitors. The morphology has an important influence on the performance of supercapacitors, and the construction of hollow, porous, and hierarchical materials should be considered in future research.

## 3. NiS-Based Electrode Nanomaterials

### 3.1. NiS/Sulfide Nanocomposites

The interface of the NiS/sulfide composite is a common interest for researchers due to the coherent properties greatly influencing the stability of the electrodes. Despite the interface modification of the sulfides, studies have implied that the morphology of the sulfides added to the NiS has a positive influence on the specific capacity. Gou et al. [100] prepared nickel sulfide using a simple hydrothermal process. The reaction temperature was adjustable for particle size and phase composition. The phase property relationship of NiS/Ni_3_S_4_ was established by means of a characterization analysis and electrochemical testing. Due to its good particle size and composition, the NiS/Ni_3_S_4_ composite delivered a preferable gravimetric capacity (194.4 mAh g^−1^ at 2 A g^−1^, and 133.1 mAh g^−1^ at 10 A g^−1^) and an impressive cycling stability (89.5 mAh g^−1^ at 10 A g^−1^ after 5000 cycles). Wu et al. [101] synthesized tubular NiS/Mo_2_S_3_ microsphere electrode materials via a solvothermal method matched with activated carbon to construct a new asymmetric supercapacitor, which improved the specific energy and cycle life of the asymmetric supercapacitor. In addition, the researchers developed a new asymmetric supercapacitor (ASC) with NiS/Mo_2_S_3_ as the cathode material and AC as the anode material, which had a higher specific energy (31.2 Wh kg^−1^) and higher specific power (850 W kg^−1^). Miao et al. [102] developed a new core–shell structure based on CoS deposited on NiS nanosheets via hydrothermal and electrodeposition methods. The core–shell structure could enhance the specific capacitance due to the larger integral area obtained. At 10 A g^−1^, the specific capacitance retention rate was 80.94% after 2000 cycles. In addition, NiS@CoS//AC asymmetric supercapacitor devices provided an energy density of 24.1 Wh kg^−1^ and significant stability (over 80% retention after 5000 cycles) at a power density of 752.15 W kg^−1^. The use of metal sulfides to construct layered core–shell structures is a unique and well-known method for further exploring the optimal adjustment and utilization of substances. The micromorphology of the composite electrodes can be optimized by adjusting the electrodeposition cycle. Xiong et al. [103] prepared a new type of lignin-free carbonized wood hybrid as an effective scaffold for loading CoS/NiS nanofibers and Co/Ni flower-like structures of cobalt and nickel nanoparticles and as an electrode material for high-performance supercapacitors. The CoS/NiS nanofibers formed some new pore structures, which was helpful for the transport of electrolyte ions. For synthetic materials, the natural porous tube structure of the wood provided a large number of electrolyte ion channels for hybrids. Flowering NiS and CoS were distributed along the carbonized wood tubes and aggregated together to form NiS nanofibers. These NiS and CoS nanofibers interwove to form new pores, which facilitated the transport of electrolyte ions. Therefore, the synergistic action of these components imparted the wood hybrid with a good supercapacitive performance: a high energy density of 46 Wh kg^−1^ (66 Wh L^−1^) and a high power density of 68 kW kg^−1^ (50 kW L^−1^). A β-NiS active material was synthesized via a one-step hydrothermal method. It was found that the introduced sulfur source had a great influence on the phase structure and morphology of Ni sulfide. A special substance (2-mercaptopropionic acid) was selected as the sulfur source to obtain pure β-NiS, and the morphological modification from microspheres to microflowers to sea-urchin-like structures was successfully achieved. The maximum specific capacity of the electrode material was 313.1 mAh g^−1^. In addition, the prepared asymmetric supercapacitor had an energy density of 49 W h kg^−1^ at a power density of 399 kW kg^−1^ and a capacitance retention rate of 98% after 5000 cycles. The electrochemical results showed that β-NiS exhibited high electrochemical activity in all morphologies, and all morphologies showed a good energy storage capacity [104]. Qin et al. [105] successfully synthesized MoS_2_/NiS yolk–shell microspheres at a low reaction temperature using a simple ionic-liquid-assisted one-step hydrothermal method. The MoS_2_/NiS microspheres had an efficient interface design and a hollow yolk–shell structure with excellent electrochemical energy storage and energy conversion performances. Further, the asymmetric supercapacitor (ASC) assembled with MoS_2_/NiS- and activated-carbon-based electrodes had a maximum energy density of 31 Wh kg^−1^ and a power density of 155.7 W kg^−1^, which showed a remarkable cycle stability and a capacitance retention rate of ~100% after 10,000 cycles.

Based on a new idea of binary synergy between sulfur sources, a high-performance α-NiS/Ni_3_S_4_ binary hybrid was successfully synthesized. A series of electrochemical results showed that this method was very effective for improving the performance of nickel sulfide. At 2 A g^−1^, the maximum specific capacity was 214.9 mAh g^−1^. In addition, the highest energy density of the assembled hybrid supercapacitor was 41.9 Wh kg^−1^ at a power density of 799 kW kg^−1^. The device had excellent cyclic stability (the capacity retention rate was 103% after 10,000 cycles) [106]. Patil et al. [107] prepared β-NiS membranes with nanoneedles, stacked nanoneedles, nanoplates, and nanoflowers via a simple anion-exchange method. The stacked nanoneedles based on β-NiS had a maximum specific capacitance (C_s_) of 415.78 F g^−1^ and an electrochemical stability of 91% after 2000 CV cycles. Nandhini et al. [108] produced a cabbage-like leaf shape on nickel foam via a simple chemical bath deposition. X-ray photoelectron spectroscopy was used to analyze the types and contents of elements in the microstructure of the materials. The results of the electrochemical properties showed that due to the clear surface morphology, fast electron conduction pathway, low charge-transfer resistance, large specific surface area, and many active sites for the electrochemical reaction, the capacitance in the specification was 1533 F g^−1^ when the current density was 7.5 A g^−1^. In addition, the composite had good cycling stability with a capacitance loss rate of only 2.1% after 3000 cycles and a maximum energy density of 35.21 Wh kg^−1^. It had a better rate performance than the electrodes based on NiS (1279.83 F g^−1^) and ZnS (616.66 F g^−1^).

**Figure 8:**
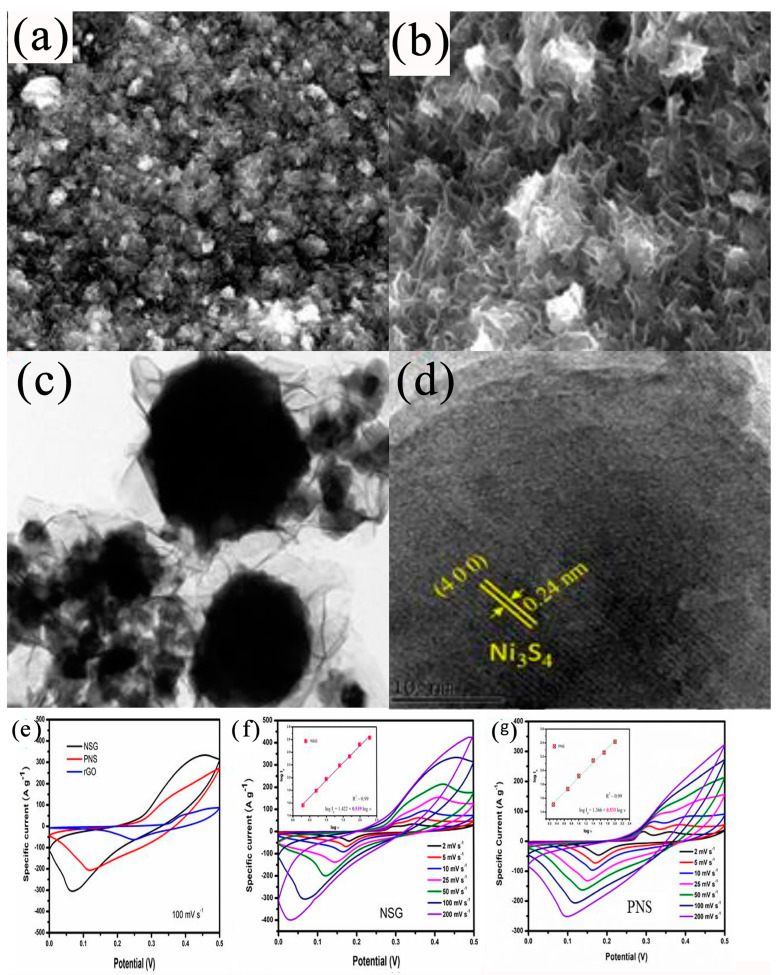
(**a**,**b**) FE−SEM images of NSG nanostructures at various nanometric scales. (**c**) TEM images of NSG nanostructure at different magnifications. (**d**) HRTEM images of NSG. (**e**) CV curves of prepared electrodes at 100 mV s^−1^. CV curves at various scan rates of (**f**) NSG and (**g**) PNS [109].

The addition of carbon materials to the electrode material also plays an immeasurable role. The study showed that the morphology of these sulfide electrode materials is essentially either granular, petal- or broccoli-leaf-like, or comprises some nanowires/nanorods. However, in the absence of additives, these electrode materials are prone to agglomeration, especially in the electrochemical charge–discharge process (that is, the process of electrons constantly cycling through adsorption and release). If there is no effect to keep them at a certain interval, the agglomeration effect is not conducive to the electrochemical process. The addition of carbon can tune the dispersity by enhancing the conductivity of the electrode’s active substance and play a role in promoting ion migration, which significantly alleviates the conduction obstruction. Carbon materials have good electrical conductivity in general, and supercapacitors have higher requirements for electrode conductivity, so carbon materials undoubtedly play an important role in improving the electrical conductivity of electrode materials. Nandhini et al. [109] synthesized graphene-coated NiS/Ni_3_S_4_ (NSG) nanostructures via a simple one-step hydrothermal method and used the encapsulated nanostructures in the design and manufacture of the supercapacitors. Compared with the original nickel sulfide, NSG had superior electrochemical properties due to its high conductivity, high specific surface area, and the synergistic effect between the graphene and nickel sulfide encapsulated nanostructures. The structural and morphological characteristics of NSG were analyzed to determine the graphene coating on the nickel sulfide. The electrochemical properties of the NSG nanostructures were then tested, which provided a high specific capacity of 827 C g^−1^ at a current density of 5 A g^−1^. After 5000 cycles, the electrodes exhibited a good cycle life (88%) and Coulombic efficiency (95%). Furthermore, the results showed that the asymmetric supercapacitor device had a high energy density of 86.3 Wh kg^−1^ and excellent cycle stability (see Figure 8).

**Figure 9:**
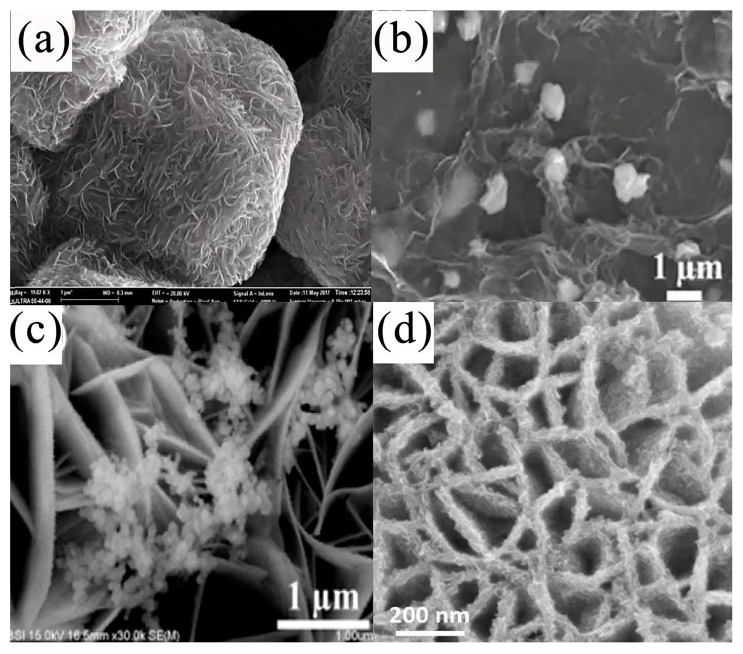
(**a**) Typical SEM images of NiS/MoS_2_ @N-rGO [112]. (**b**) SEM images of Ni_3_S_4_@rGO-20 [113]. (**c**) SEM images of NiO/NiS nanosheets with nanoparticles on Ni foam [114]. (**d**) SEM images of NiS/SnS_2_@CC [114].

To obtain highly cost-effective composite metal sulfides, Subramanian et al. [110] coated metal sulfides (such as nickel sulfide and cobalt sulfide) on foamed nickel hierarchical plates so that the PEDOT:PSS could be evenly distributed on the bases of the plates. At a 15 mA cm^−2^ current density, the maximum specific capacitance of the NiS/CoS/PEDOT:PSS composite electrodes was 353 F g^−1^ while the capacitances of the NiS/CoS and NiS electrodes were 313 F g^−1^ and 182.7 F g^−1^, respectively. After 1000 cycles, the electrodes obtained a longer stable cycle curve with an initial capacitance retention of 92.82%. These electrochemical results showed that PEDOT:PSS coated on the NiS/CoS/foamed nickel improved the conductivity and electrochemical properties compared with the NiS/CoS and NiS electrodes. A novel 3D NiS/MoS_2_@N-doped hybrid composite with reduced GO was synthesized via a simple and inexpensive hydrothermal method. Mesoporous nanostructures were prepared via the combination of nickel sulfide and molybdenum disulfide with N-reduced graphene oxide, which increased the approachable area of the electrolyte ions. The use of N-rGO during the preparation process reduced the chance of NiS and MoS_2_ aggregating at the interface, which thereby created a synergistic effect between NiS and MoS_2_. Scanning electron microscope images showed clearly that the composite materials looked like nanoflowers and had a good dispersed structure. Therefore, the NiS/MoS_2_@N-rGO hybrid exhibited an excellent capacitance performance, and its ultrahigh ratio was synergistic with this 3D network. When the current density was 1 A g^−1^, the mixed NiS/MoS_2_@N-rGO electrodes showed a high specific capacity. A fully symmetric NiS/MoS_2_@N-rGO supercapacitor was also tested; the test results indicated that it had a good electrochemical performance. More importantly, the symmetric supercapacitors had good flexibility under different bending conditions [111] (see Figure 9a). Huc et al. [112] synthesized a Ni_3_S_4_@RGO material in situ via a one-step hydrothermal method using the unique sulfur source 2-mercaptopropionic acid and GO as an inducer. It was found that graphene oxide (GO) induced the oxidation of Ni^2+^ to Ni^3+^ during the hydrothermal process, and the morphology of the NiS/Ni_3_S_4_ microrods was transformed into a multihedral Ni_3_S_4_ morphology. The effects of the GO content and oxidation degree on the phase composition and morphology of nickel sulfide were studied. It was concluded that graphene’s oxygen-containing functional groups facilitated the transformation process. The GO content and oxidation degree played a key role in the synthesis of high-purity Ni_3_S_4_ (see Figure 9b). Through a simple two-step hydrothermal route, Sang-Yong Kim et al. [113] successfully synthesized highly cost-effective NiO nanosheets coated with NiS nanoparticles on nickel foam. The high conductivity, large surface area, and unique surface morphology of NiO/NiS provided a fast ion diffusion rate and a fast electron transfer rate for the obtained NiO/NiS hybrid electrodes. Therefore, the composite NiO/NiS electrode material exhibited a high specific capacitance and good cycle stability of 386.7 F g^−1^ at a current density of 1 A g^−1^. At 5 A g^−1^, and the capacitance could still be maintained at 97.6% after 3000 charge–discharge cycles (see Figure 9c). Guan et al. [114] prepared porous NiS/SnS_2_ core–shell heterogeneous nanowall arrays on carbon cloth via simple cation exchange using SnS_2_ nanochip arrays as precursors. Due to the maximum utilization of active materials and the synergistic effect between the SnS_2_ core and NiS shell, the electrodes showed a larger specific surface area, which maximized the utilization of active materials and enhanced the reaction kinetics. The electrodes had a good multiplier performance of 185.08 mAh g^−1^ at a current density of 40 mA cm^−2^ (23.26 A g^−1^) and a satisfactory cycle stability (the capacity retention rate still reached 82.6% after 1000 cycles; see Figure 9d).

In addition to the above review, there are some electrode materials made from simple composites with nickel sulfide. Due to the high performance of NiS electrodes, other sulfides—including CrS, MoS_2_, and CoS—have been added by researchers to construct different morphologies with various interfaces. Cheng et al. [115] prepared α-NiS/CrS composites, and the specific capacitance of the electrode material was calculated. The electrode showed a high capacitance of 1092 F g^−1^ at 1 A g^−1^, and the structure of the α-NiS/CrS hybrid product remained stable over 2000 cycles, proving that this unique structure could effectively prevent active nuclear fusion and improve the aggregation of active nuclear fusion and thereby achieve good cycle stability. NiCo_2_S_4_/NiS hollow nanospheres with excellent electrochemical properties were synthesized via a two-step hydrothermal method [116]. Ni and Co can lead to multiple redox reactions and change the morphology of the NiS electrode, which is beneficial to the high capacitance. The asymmetric supercapacitor achieved a good performance of 43.7 Wh kg^−1^ at a 160 W kg^−1^ power density. When the current density was 1 mA cm^−2^, the specific capacitance was 123 F g^−1^. After 3000 cycles, the capacitance degraded by 5.2%. In addition, the NiCo_2_S_4_/NiS electrodes had a specific capacitance of 1947.5 F g^−1^ at 3 mA cm^−2^ and remained 90.3% stable after 1000 cycles. Asgharet et al. [117] synthesized nickel sulfide, zinc sulfide, and their composite materials via a surfactant-driven hydrothermal method. At a scanning rate of 5 mV s^−1^, the maximum specific capacitance of the NiS/ZnS composite was 1594.68 F g^−1^. After 3000 charge–discharge cycles, the retention rate of the composite material was 95.4%.

Sulfur is less electronegative than oxygen. Sulfides have a more flexible structure, which makes electron transport easier. Despite the interface changes caused by the combination of sulfide and NiS, studies have shown that the morphology of sulfide and the use of some special means of doping carbon materials or other chemical methods have positive effects on the optimization of the electrochemical performance of the NiS electrode material, such as increasing the specific capacitance value and enhancing its conductivity.

### 3.2. NiS/Carbon Nanocomposites

At present, the development of new electrode materials with a high energy density, high power, high stability, and good safety performance has become an important means by which to improve the performance of supercapacitors and lithium-ion batteries. According to a large number of important documents, NiS materials have problems such as poor conductivity, the slow diffusion of ions in the material, a small active area, and a large change in the volume of lithium-ion insertion and removal when used as electrode materials in practical applications, which leads to their inability to meet actual needs and limits their extensive application in industrial production. In order to solve these problems, it is still a challenge to find better electrode materials for supercapacitors. Nanocomposites play an important role in improving electrochemical activity, which can greatly improve their unique physical and chemical properties. To date, a large number of researchers have committed to optimizing the design of NiS nanocomposites through the combination of carbon materials and other transition metal oxides.

Carbon materials have many advantages, such as their diverse structures, rich surface states, good chemical stability, and strong controllability. In addition, they have excellent electrical transport properties and highly active surfaces. They are key components in electrochemical energy storage systems. They are used in electrochemical energy storage devices/systems in various forms, such as active substances, conductive agents, coatings, flexible substrates, and electrocatalytic carriers, and play an important role. In particular, new carbon nanomaterials—such as graphene and carbon nanotubes—have excellent conductivity, a high specific surface area, and the ability to build three-dimensional network structures [118]. Therefore, researchers have chosen carbon materials as the preferred high-performance composite materials. Carbon materials are ideal materials for the composite electrodes of supercapacitors. They show great potential in the field of electrochemical energy storage and have been developed rapidly in recent years. In order to improve the performance of supercapacitors, a variety of nanostructured electrode materials have been prepared, including carbon nanotubes, carbon nanofibers, graphene, biomass-derived carbon materials, and other nanostructures.

#### 3.2.1. NiS/Carbon Nanotube Composites

**Figure 10:**
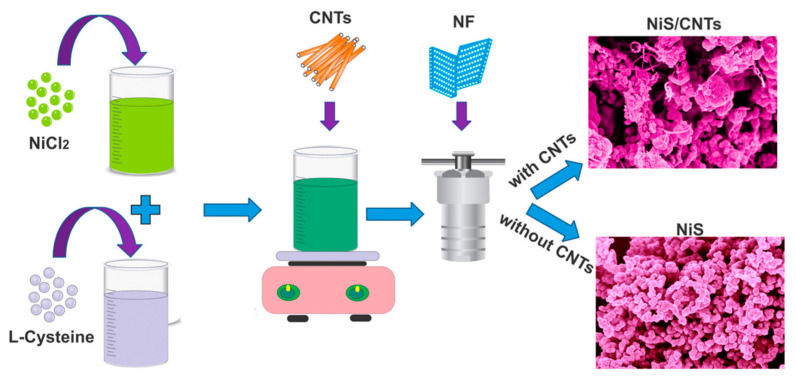
Scheme for the preparation of NiS@NF and NiS/CNTs@NF electrodes [119].

NiS/CNTs can be loaded to nanohybrids on three-dimensional nickel foam (NF) with a self-supporting design (see Figure 10). In a study by Sabeeh et al. [119], the NiS/CNTs@NF electrode had a larger surface area, self-supporting design, and better conductivity. The electrode material only lost 4.9% of its capacity after 3000 continuous constant-current galvanostatic charge–discharge (GCD) cycles, and it had a good cycle performance and activity. In addition, although the current density was increased by fivefold, the mixed electrode could maintain a capacity of ~84.5%. The impedance test results showed that the electrochemical reaction between the NiS/CNTs and the electrolytes was faster and more reversible.

Ouyang et al. [120] found that the combination of battery materials and carbon materials is of great importance for solving the problem of particle aggregation. Hexagonal nickel sulfide has been widely studied and applied to supercapacitors because of its high theoretical capacitance, simple synthesis process, and low cost; however, its poor conductivity and easy caking seriously restrict its practical application. NiS nanoparticles and activated carbon nanotubes (NiS/ACNTs) with abundant active groups could effectively inhibit the aggregation of NiS nanoparticles and showed good electrochemical properties after hydrothermal methods and an annealing treatment were used. The specific capacitance of the hybrid electrodes was up to 1266 F g^−1^ at a current density of 1.0 A g^−1^ and 1028 F g^−1^ at 10 A g^−1^ (81% of the initial specific capacitance). In addition, asymmetric supercapacitors (ASCs) with NiS/ACNTs as the positive pole and activated carbon (AC) as the negative pole had a high energy density of 36.0 Wh kg^−1^ at a power density of 806 W kg^−1^ and a capacity retention rate of 83% at 2.0 A g^−1^ after 2000 cycles. Yang et al. [121] prepared nickel sulfide/MOS (NMS/CNT) one-dimensional hierarchies supported by carbon nanotubes (CNTs) using a glucose-assisted hydrothermal method. The coiled and entangled one-dimensional structures intertwined with one another to construct a three-dimensional (3D) porous network that facilitated the entry of electrolytes. In addition, the formation of the metal 1T-2H hybrid MoS_2_ and the synergistic effect between the MoS_2_ layer and the nickel sulfide (NS) nanoparticles promoted the diffusion of ions on the electrode surface. The volume change during the redox process could be tolerated due to the void space formed between the NMS sheets, which made the electrode material structure more stable. Thus, the assembled NMS/CNT//activated carbon (AC) asymmetric supercapacitor had a good specific capacitance of 108 F g^−1^ at 0.5 A g^−1^ while having a high energy density of 40 Wh kg^−1^. After 10,000 cycles of charge and discharge, an excellent cycle stability of almost 100% capacity was achieved.

#### 3.2.2. NiS/Carbon Nanofiber Composites

**Figure 11:**
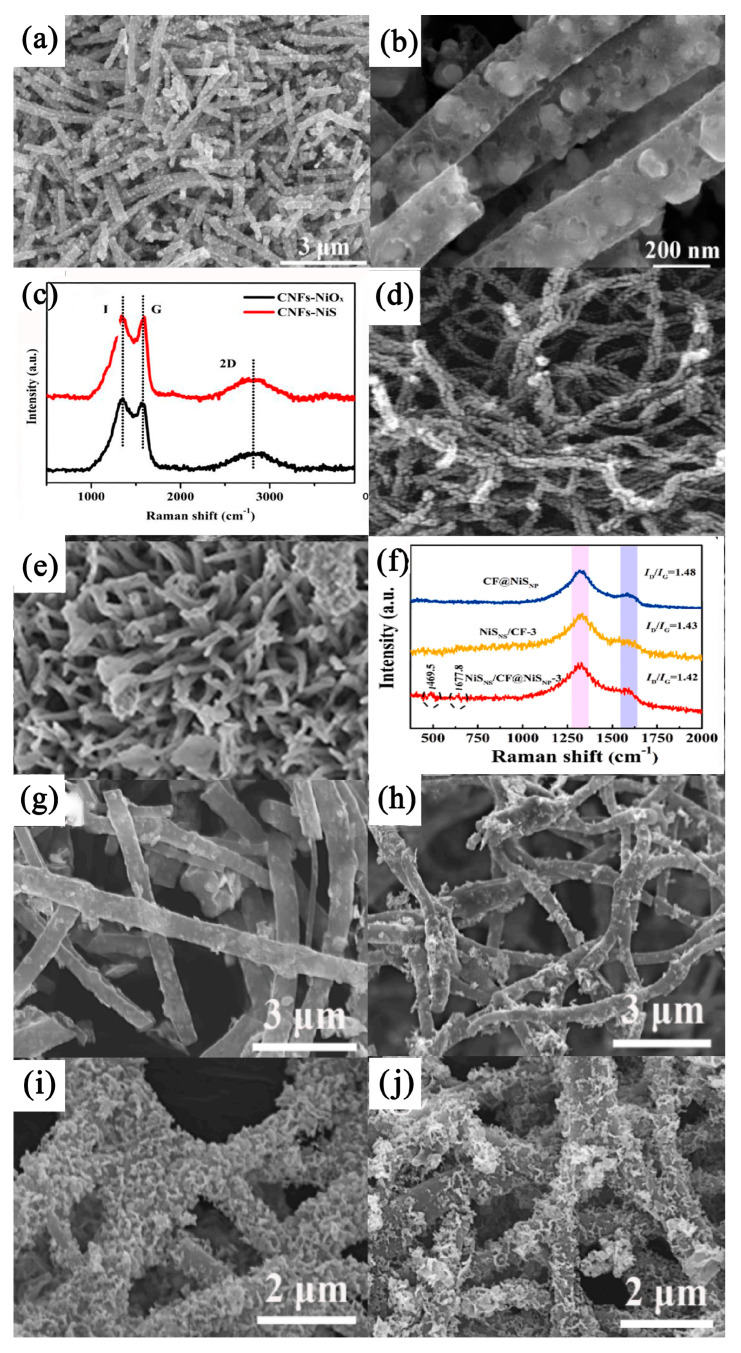
SEM images of (**a**,**b**) CNFs-NiS, (**c**) Raman spectra of CNFs-NiO_x_ (black) and CNFs-NiS (red) [123]. (**d**) CNTs/NiS, (**e**) CNTs/NiS/CoS [124], (**f**) Raman spectra of NiS/CF@NiS, (**g**) NiS_NF_/CF@NiS_NP_-1, (**h**) NiS_NF_/CF@NiS_NP_-2, (**i**) NiS_NF_/CF@NiS_NP_-4, and (**j**) NiS_NF_/CF@NiS_NP_-5 [125].

Carbon nanofiber (CF) is a kind of inorganic carbon material that is generally in the shape of a fiber and in which the carbon mass fraction is usually more than 90%. Carbon nanofibers (or carbon fibers with nanoscale dimensions), like other one-dimensional nanostructures such as nanowires, nanotubes, and single-molecule wires, have received increasing interest because of their extremely high aspect ratios. Activated carbon nanofibers (ACFs) are special carbon materials with a high specific surface area and are obtained by activating carbon nanofibers. They are also representative among many carbon-based materials. They have a high specific surface area (up to 3000 m^2^ g^−1^) and a relatively controlled aperture size distribution [122]. Xu et al. [123] suggested that the close relationship between graphite carbon and NiS nanoparticles could be attributed to the accessible specific surface and unique porous structure of CNFs, as well as the high specific volume of CNFs-NiS. The CNFs-NiS composite that they studied was prepared via the electrospinning, calcination, and in situ vulcanization of NiS nanoparticles wrapped with graphite carbon nanofibers (see Figure 11a,b). Two broad peaks at 1350 cm^−1^ and 1580 cm^−1^ were assigned to the typical D and G bands of carbon, respectively. The I_D_/I_G_ values of CNFs-NiO_x_ (1.42) and CNFs-NiS (1.38) decreased, which was attributed to the loss of low-graphite carbon due to high-temperature vacuum calcination (see Figure 11c). The CNFs-NiS electrode had a specific capacity of 177.1 mAh g^−1^ (0.41 mAh cm^−2^ at 2.3 mA cm^−2^ current density) at a current density of 1 A g^−l^ and had a high long-term cycle stability. After 5000 cycles, the capacitance retention rate was 88.7%. In addition, the asymmetric supercapacitor had an enhanced energy density capacity and high cycle stability. After 5000 cycles, the capacitance retention rate was 89.5%.

Carbon nanotubes/nickel sulfide/cobalt sulfide (CNTs/NiS/CoS) nanocomposites are widely used in the field of electrode materials for supercapacitors because of their low cost and good electrochemical performance. Studies have shown that CNTs/NiS/CoS can significantly reduce the overall impedance, which might be one of the key factors in improving the electrochemical performance of CNTs/NiS/CoS (see Figure 11d,e). In a study by Xavier et al., when the current density was 1 A g^−1^, the specific capacitance of the CNTs/NiS/CoS electrode remained at 97.17% after 8000 charge–discharge cycles. Its energy density and power density were 624.44 Wh kg^−1^ and 8325.87 W kg^−1^, respectively. The CNTs/NiS/CoS nanocomposites showed a high specific capacitance and long cycle life under constant-current charge–discharge measurements [124]. However, in practical production, the specific capacitance of carbon fiber was always very low due to the insufficient utilization of its structure, which seriously hindered its application in high-performance supercapacitors. Some researchers have put forward a concept of “maximizing the utilization of structure” and successfully prepared interconnected NiS_NF_ through electrospinning and impregnation vulcanization with CF@NiS_NP_ composite materials. Compared with the I_D_/I_G_ values of CF@NiS_NP_ (1.48) and NiS_NF_/CF-3 (1.43), NiS_NF_/CF@NiS_NP_-3 possessed the smallest I_D_/I_G_ value of 1.42, indicating the highest degree of graphitization, which is essential for improving electrical conductivity and ensuring effective electrochemical responses. This electrode material showed a high reversible specific capacitance, high energy density, and a long cycle life, and effectively expanded the structural utilization of carbon fiber composites [125] (see Figure 11f–j).

#### 3.2.3. NiS/Carbon–Graphene and Biomass-Derived Carbon Composites

In the world of carbon nanomaterials, in addition to the known magic of carbon nanotubes and fullerenes, two-dimensional materials have attracted extensive attention due to their unique physical, chemical, and electrochemical properties. Two-dimensional functional materials such as graphene have become very popular in recent years. Graphene has a unique two-dimensional planar honeycomb structure and can be used as a basic component of zero-dimensional, one-dimensional, and three-dimensional carbon materials. In fact, graphene is the basic unit (i.e., basic component) that constitutes carbon nanotubes, fullerenes, and even graphite blocks. Today, crystals composed of carbon atoms with several atomic layers (usually fewer than 10 layers) can also be called graphene. Graphene has a high theoretical specific surface area of ~2630 m^2^ g^−1^, and its theoretical electrochemical double-layer capacitance is up to 550 F g^−1^. Graphene is considered to be an ideal electrode material for supercapacitors due to its excellent electrical conductivity and mechanical properties.

Zhu et al. [126] recorded the preparation of NiS@NC-300 and NiS@NC-500 NiS core–shell composites coated with nitrogen-doped carbon at different temperatures (300 and 500 °C). This core–shell structure helped to buffer the volume change in NiS during charge–discharge cycles. In addition, the carbon layer was tightly connected with NiS nanoparticles and formed a stable interconnected network framework and enhanced the electrochemical stability. Due to these advantages, at 0.5 A g^−1^, the specific capacity was 665 C g^−1^ (1330 F g^−1^) and the cycle stability was significantly improved. At 10 A g^−1^, 92.3% of the initial capacitance was retained in 3000 cycles, which was much better than NiS from NiS@NC-300. Asymmetric supercapacitors consisting of commercial activated carbon (AC) electrodes provided a high energy density of 28.6 Wh kg^−1^ at 884.5 W kg^−1^. They had good cycle stability and retained 81.7% capacitance after 3000 cycles. NiS/rGo electrode materials can improve the conductivity and volume expansion of NiS during charge–discharge cycles, as well as the conductivity of graphene. However, due to its complex preparation process, this is time consuming and consumes a high amount of energy, and these issues still need to be solved. 

**Figure 12:**
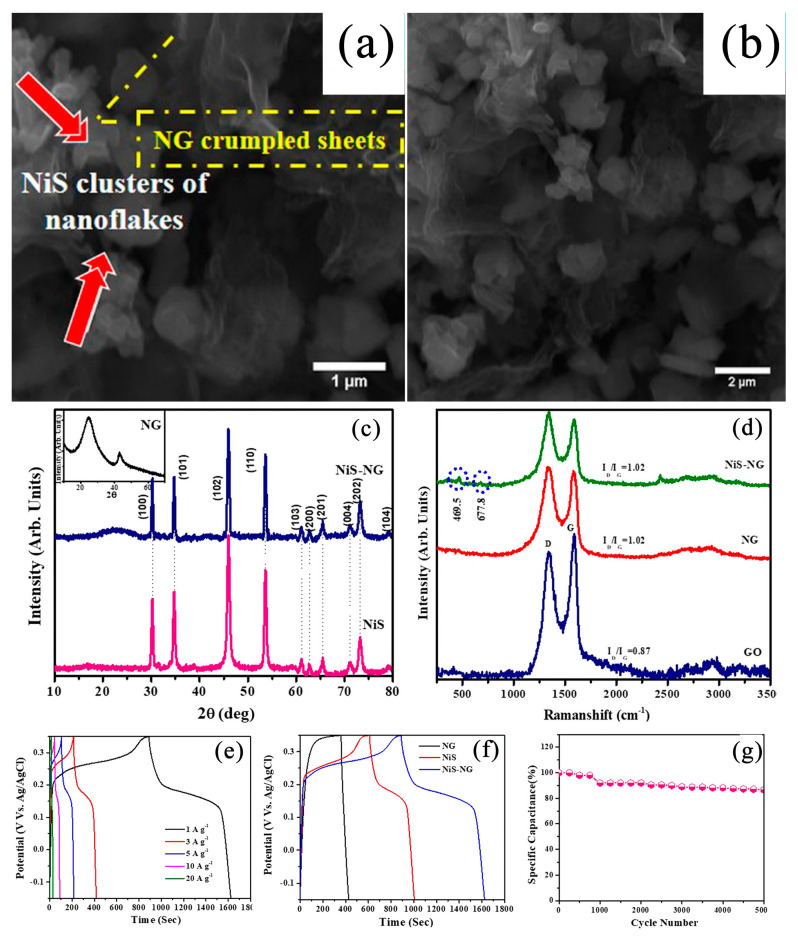
(**a**,**b**) SEM morphologies of NiS–NG. (**c**) XRD and (**d**) Raman profiles for NiS–NG. Galvanostatic charge–discharge studies: (**e**) discharge plots of NiS–NG; (**f**) comparative GCD plots for NG, NiS, and NiS–NG. NG//NiS–NG asymmetric device performance: (**g**) cyclic stability [127].

Using a simple and green microwave synthesis method to prepare nitrogen-doped graphene via a hydrothermal method, Reddy et al. [127] prepared NG/NiS nanocomposites as electrode materials for supercapacitors. The prepared materials were characterized via XRD, Raman, XPS, SEM, EDX, and element mapping characterization techniques to obtain information on their structure, composite formation, morphology, and elemental contents. The material structural information with respect to NG, NiS, and NiS-NG was confirmed via XRD. In order to better study graphene-based materials, Raman analysis was used to test them, and the reduction effect and the formation of composite materials were also proven. XPS was used to describe the properties of the composites and to reveal information about their chemical composition and formation. SEM images of NiS-NG revealed the formation process of the NiS-NG composite: NiS gradually opened into nanoclusters from nanoflakes. The EDX and element mapping of NiS-NG supported the other results of the SEM analysis. Electrochemical studies such as CV, GCD, and impedance analysis showed that NG/NiS was a high-quality electrode material with a specific capacitance of 1467.8 F g^−1^ at 1 A g^−1^. In addition, the asymmetric structure of an NG//NG/NiS battery was designed. The energy density was 66.66 Wh kg^−1^ and the power density was 405.83 W kg^−1^. Even after 5000 charge–discharge cycles, the cycle performance could still reach 86.6% (see Figure 12).

**Figure 13:**
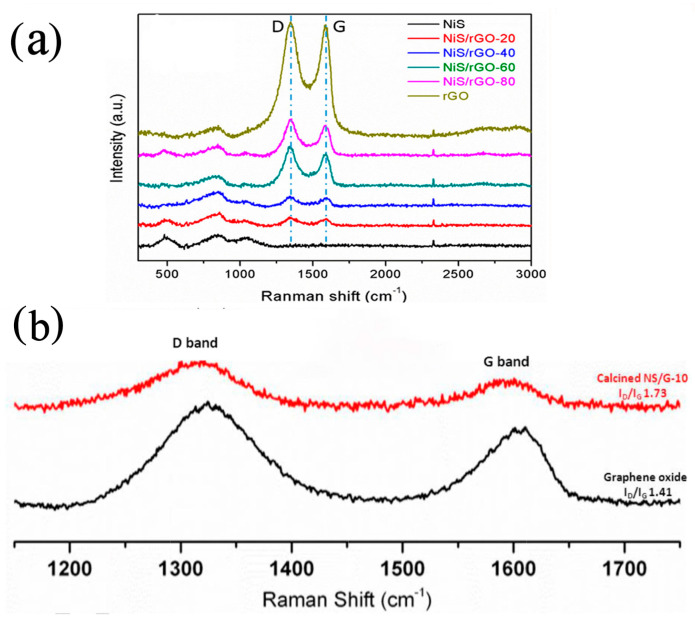
(**a**) Raman spectra of the NiS, NiS/rGO−20, NiS/rGO−40, NiS/rGO−60, NiS/rGO−80, and rGO samples [128]. (**b**) Raman spectra of GO and calcined NS/G−10 [130].

Wang et al. [128] synthesized NiS/rGo hybrid electrode materials via a new microwave–hydrothermal one-step method. The preparation time was significantly shortened from a few days to only six hours without using any chemical surfactants. The prepared NiS/rGo hybrid had a low resistance of 0.48 Ω. It had a very high specific capacitance (1745.67 F g^−1^ at 1 A g^−1^) and a high cycle retention rate (451.21 F g^−1^ at 10 A g^−1^ after 3000 continuous charge–discharge cycles). The symmetrical solid-state supercapacitor assembled with this NiS/rGo hybrid electrode material had a specific capacitance of 14.20 F g^−1^, a high energy density of 7.1 Wh kg^−1^, and a high power density of 1836 W kg^−1^, which shows great potential for application in the field of energy storage devices. The rGO in the hybridization process was represented by a Raman spectrum (see Figure 13a).

Li et al. [129] used three-dimensional macroporous delamination via immersion drying and electrodeposition to prepare MCS@GNS@NiS composite materials as electrode materials for high-performance supercapacitors. Using commercial flexible reinforcing cotton (MCS) as a framework, a uniform three-dimensional interconnected graphene-coated macronetwork was constructed. The porous NiS film modified by nanoparticles providing a higher electrode–electrolyte contact area and a shorter ion diffusion path, which accelerated the charge-transfer reaction. The results showed that the MCS@GNS@NiS composites had a specific capacitance of 775 F g^−1^ at a charge–discharge-specific current of 0.5 A g^−1^. After 1000 cycles at a current density of 2 A g^−1^, the capacitance retention rate was 88.1%. In addition, the MCS@GNS@NiS electrode was able to provide a high energy density of 11.2 Wh kg^−1^ even under a high power density of 1008 W kg^−1^. 

AbdelHamid et al. [130] synthesized nickel sulfide and graphene-coated nickel sulfide nanosheets, which significantly improved the conductivity of nickel sulfide supercapacitors. As a supercapacitor electrode, the specific capacitance of the nickel sulfide nanosheets reached more than 1000 F g^−1^ at a current density of 5 A g^−1^, which represents one of the best results with respect to the research on nickel sulfide electrode materials to date. At a high current density, the introduction of graphene significantly improved the specific capacitance of nickel sulfide (see Figure 13b). The intensity of the D band to G band ratio (I_D_/I_G_) was increased by the graphitization of the sample. It was found that the I_D_/I_G_ ratio increased from 1.41 in GO to 1.73 in NS/G-10, which may have been due to the decrease in the amount of GO, which generated defects.

A hybrid supercapacitor device was successfully constructed by synthesizing an NCD-modified CO-NiS flower-like structure using an effective two-step solvothermal method and coupling it with a PPD-modified reduced graphene oxide (rGO) anode. The electrochemical results showed that the device had a high energy density of 71.6 Wh kg^−1^ at 712.0 W kg^−1^ and good cycle stability (78.3% after 12,000 cycles at a current density of 5 A g^−1^) [131]. Sun et al. [132] synthesized a carbon thermal oxide self-doped graphite carbon nitride/NiS (TC-g-C_3_N_4_/NiS) composite by combining nickel sulfide with carbon self-doped carbon nitride via a hydrothermal method. The NiS particles uniformly adhered to the g-CN layer to avoid particle aggregation. The prepared composites maintained a high specific capacitance (1162 F g^−1^) when the current density was 1 A g^−1^, and they showed good cycle stability (the capacitance retention was 82.0% after 8000 cycles). In addition, the assembled TC-g-C_3_N_4_/NiS//AC had a high energy density (27 Wh kg^−1^) and good cycle performance (the capacity retention rate was 87.9% after 8000 cycles).

In recent years, high-quality activated carbon from renewable carbon resources (such as biological waste [133], mango seed shells [134], eggshells [135], grape slag [136], etc.) has been studied. Biowaste eggshells can be used as cathodes in their calcined state and have been found to be suitable as anodes for electrochemical cells. This not only allows for the reversible storage of energy, but also for waste management and sustainable development goals by diverting materials from landfills. Minakshi et al. explored the feasibility of eggshell-derived materials as cathodes/anodes for batteries and capacitors. Electrochemical studies showed that both electrodes exhibited quasi-box-shaped potentiostatic curves, indicating that they have the characteristics of capacitors. The CaCO_3_ cathode had a medium discharge capacitance of 10 F g^−1^ while the CaO anode had a high capacitance of 47.5 F g^−1^. Under the condition of a 0.15 A g^−1^ current density, the CaO electrode showed 55 F g^−1^ in both the positive and negative regions, and the retention rate was close to 100% after 1000 cycles. The authors also used grape residue as a raw material to prepare porous carbon through carbonization and chemical activation, which also achieved good results in the field of energy storage after tests and calculations. These findings all demonstrate the technical importance of energy storage applications and may help in the reassessment of biological waste prior to disposal.

Biomass carbon can be easily obtained, and its morphology can vary significantly; however, graphene generally displays a uniform nanosheet structure that is useful for the accurate design of highly conductive and cyclically stable electrodes. Moreover, graphene could be beneficial for flexible electrode construction. The main shortcoming of graphene may be the complexity of its production process and the high cost compared to biomass-derived carbon.

#### 3.2.4. NiS Composites with Other Carbon Nanomaterials

Carbon microspheres are a zero-dimensional carbon material with a diameter of 100 nm~1 μm and with a cage structure and regular morphology; additionally, they have the outstanding advantages of having good conductivity, thermal conductivity, a large specific surface area, and excellent chemical and thermal stability. Using biological yeast as the carbon parent template, Zhang et al. [137] designed and synthesized a new type of NiS/CMS binary hybrid microsphere. It was found that these NiS/CMS binary hybrid microspheres had a specific capacitance of 1594 F g^−1^ at 1 A g^−1^, and the hybrid supercapacitor (HSC) had a high energy density and power density, as well as high cycle stability. Wu et al. [138] used an in situ hydrothermal method to deposit NiS nanoparticles on porous hollow carbon spheres (PHCSs) to form a three-dimensional lychee-shell-like double-shell structure. PHCSs effectively inhibited the aggregation of NiS nanoparticles, ensured more storage sites, and improved the performance of the electrode materials. In addition, the assembled hybrid device provided a high energy density of 24.4 Wh kg^−1^ at a power density of 767 W kg^−1^ and retained an 89.3% capacity after 5000 ultralong charge–discharge cycles.

Nickel–sulfur and multidimensional carbon nanomaterial composites have also shown good test results in scientific research. Zhang et al. [139] obtained hexaphase NiS octahedrons via phase transformation and reduced graphene oxide via a multidimensional carbon comodification to 0D carbon quantum dots, 1D carbon nanotubes, and 2D reduced graphene oxide (NiS@CQDs-CNTs-rGO). At a current density of 20 A g^−1^, the capacity was 149 mAh g^−1^, which was better than that of the other phases: NiS@CNTs-rGO (154 mAh g^−1^ at 1 A g^−1^, 52 mAh g^−1^ at 20 A g^−1^) and Ni_7_S_6_@CNTs-rGO (167 mAh g^−1^ at 1 A g^−1^, 124 mAh g^−1^ at 20 A g^−1^). In addition, asymmetric supercapacitors (ASCs) assembled from NiS@CQDs-CNTs-rGO and graphene hydrogel achieved remarkable cycle stability (82% capacity after 5000 cycles). The XPS results showed that there was a strong C–S bond between the carbon matrix and the NiS NPs, which improved the structural stability and ensured good long-term cycle stability. The improvement in electrochemical performance was mainly attributed to the 0D, 1D, and 2D carbon structures, as well as the strong C–S bonds between the active substance and the carbon matrix.

Zero-dimensional carbon material has a large specific surface area and can more easily reach the electrode surface than one-dimensional carbon material electrolytes and active materials. However, due to the introduction of a binder in the fabrication process of a zero-dimensional material, its conductivity is low. One-dimensional carbon material has a conductive nanolinear structure and can deposit various electroactive materials. The composites of carbon materials with different dimensions can improve the electrical conductivity, increase the specific surface area, enhance the mechanical properties, and improve the stability of the capacitor performance, which is a feasible direction for the construction of supercapacitors in the future. The three-dimensional structure of two-dimensional graphene can be loaded with active nanostructures such as zero-nanometer particles, one-dimensional nanotubes, nanowires, two-dimensional nanosheets, etc. The composites of multistage materials can combine the advantages of the structure and properties of carbon materials of different dimensions so as to prepare electrode materials for supercapacitors with better performance.

The results of the abovementioned research show that the combination of transition metal sulfides and carbon materials as positive electrode materials produces an excellent pseudocapacitance performance, significantly improves the conductivity and electrochemical reactivity of the electrode materials, and improves the storage capacity, energy density, power density, and cycle stability of the supercapacitors. These characteristics have important guiding significance for the design of new electrode materials and the development of high-performance supercapacitors. Biomass-derived carbon shows various advantages, and NiS-loaded composites are widely studied; however, how to precisely control the morphology of biomass-derived carbon materials merits further in-depth study.

### 3.3. NiS/Transition Metal Oxide Nanocomposites

**Figure 14:**
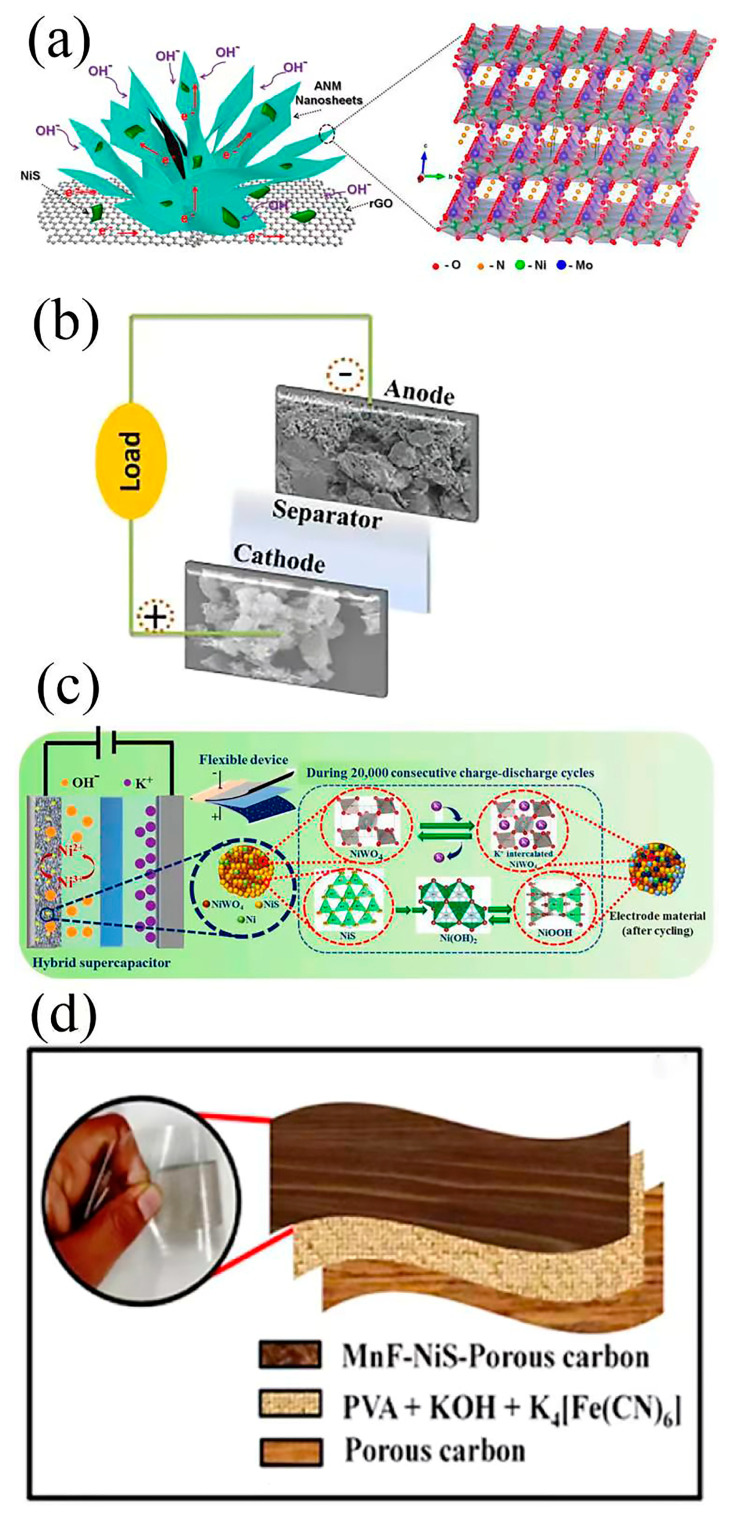
(**a**) Schematic representation showing the possible working mechanism of ANM-NiS- rGO as a supercapacitor electrode [140]. (**b**) Schematic diagram of an ASC [141]. (**c**) Schematic diagram of an ASC [146]. (**d**) Schematic representation of a fabricated all-solid-state flexible asymmetric supercapacitor cell [147].

It is well known that O vacancies can enhance electronic transmission, and oxides can be easily made with O vacancies in the materials; this is one important aspect in the design of NiS/transition metal oxide nanocomposites. Moreover, the oxide can change to other phases during the electrochemical process, and the new phase could improve the interface state. Moreover, transition metal oxides are the most common redox-active materials and are used as electrode materials for SCs—mostly asymmetric—specifically in oxide and hydroxide forms. Ke et al. [140] synthesized a new ternary composite (ANM-NiS-rGO) consisting of ammonium nickel molybdate, nickel sulfide, and reduced graphene oxide via a two-step hydrothermal method (see Figure 14a). The morphological analysis showed that the reduced graphene oxide nanosheets in the ternary composites were modified by electrochemically active NiS nanoparticles and ANM microflowers. The ternary composite had a porous three-dimensional flower-like structure with a large surface area of 135 m^2^ g^−1^ and a high pore volume of 0.258 cm^3^ g^−1^. However, when ANM NiS rGO was used as the positive pole of a battery-type hybrid supercapacitor, its specific capacity was 150 mAh g^−1^ when the current density was 1 A g^−1^. The results showed that the ternary composites had a higher specific surface area and conductivity, higher specific capacity, and better rate performance and cycle stability. In order to verify their practical application, hybrid supercapacitors were prepared with ANM NiS rGO and rGO as the positive and negative electrodes, respectively. The device worked normally in the voltage range 0–1.75 V and it showed a high specific capacity of 28 mAh g^−1^ at a current density of 2 A g^−1^. It had a maximum energy density of 24.2 Wh kg^−1^ at a power density of 1.75 kW kg^−1^. Guo et al. [141] grew interconnected NiS/Co_3_S_4_ nanosheets on an Fe_2_O_3_ substrate (Fe_2_O_3_@NiS/ Co_3_S_4_) via a simple in situ solution vulcanization process (see Figure 14b). Fe_2_O_3_-based cathode materials produced in this way are not ideal due to their poor conductivity, rate capacity, and long-term cycle stability, among other problems. Due to the synergistic effect between Fe_2_O_3_ nanoparticles and NiCo-S nanosheets, as well as their unique three-dimensional structure—which provide abundant channels and active sites—the electrochemical properties of Fe_2_O_3_ nanoparticles as positive electrode materials for supercapacitors have been studied. The specific capacity of the electrode material was 1213.7 F g^−1^ at 1 A g^−1^, and the capacity retention rate was 85.2% after 5000 cycles at 5 A g^−1^. The asymmetric supercapacitor was assembled with Fe_2_O_3_@NiS/Co_3_S_4_ as the positive electrode and activated carbon as the negative electrode. At 5 A g^−1^, the capacitance retention rate was 92.4%. Zheng et al. [142] used the one-step microwave method to make NiO/NiS@CNT nanocomposites. The specific capacitance at 1 A g^−1^ was 809.7 F g^−1^, and the cycle stability at 5 A g^−1^ was very high. The retention rate after 20,000 cycles was ~100%. Asymmetric supercapacitor devices based on NiO/NiS@CNT//active carbon (AC) exhibited high energy, a high power density, and good cycle stability. As electrode materials for supercapacitors, heterogeneous (NiO)_0.1_(NiS)_0.9_ multishell hollow microspheres exhibited the best electrochemical properties in a range of nickel oxide/sulfide compounds. The (NiO)_0.1_(NiS)_0.9_ sample maintained a high specific capacitance of 486 F g^−1^ after 10,000 cycles, even at a high current density of 50 A g^−1^. In addition, the (NiO)_0.1_(NiS)_0.9_‖ activated carbon asymmetric supercapacitor (ASC) had a specific capacitance of 58.5 F g^−1^ at 2 A g^−1^ in a 2 M KOH solution. This excellent electrochemical performance could be attributed to the careful composition regulation and stable multishell structure [143].

Porous NiS nanoflake arrays were successfully synthesized via a facile solution-based ion-exchange reaction (IER) method based on preformed chemical bath deposition (CBD)-derived NiO nanoflake arrays. The NiS nanoflake arrays preserved the morphology of precursor–NiO nanoflake arrays and grew vertically to the substrates. As the cathodes of supercapacitors, they exhibited outstanding electrochemical performances with a high specific capacitance (718 F g^−1^ at 2 A g^−1^) and good cycle performance and capacity retention (593 F g^−1^ after 3000 cycles at 2 A g^−1^) [144]. Wu et al. [145] synthesized porous NiS/CoO nanosheet composite arrays on nickel foam via one-step electrodeposition and used them as electrode materials for supercapacitors, which showed a high specific capacitance of 1054 F g^−1^ at a high current density of 6 A g^−1^ and good long-term cycle stability. The unique layered porous structure of the treatment increased the surface area and gap, which was conducive to charge-transfer and redox reactions. The high capacitance of the hybrid electrode was due to the synergistic action of NiS and CoO, and the enhanced contact between the active material and the substrate interface imparted the electrode with good conductivity.

**Figure 15:**
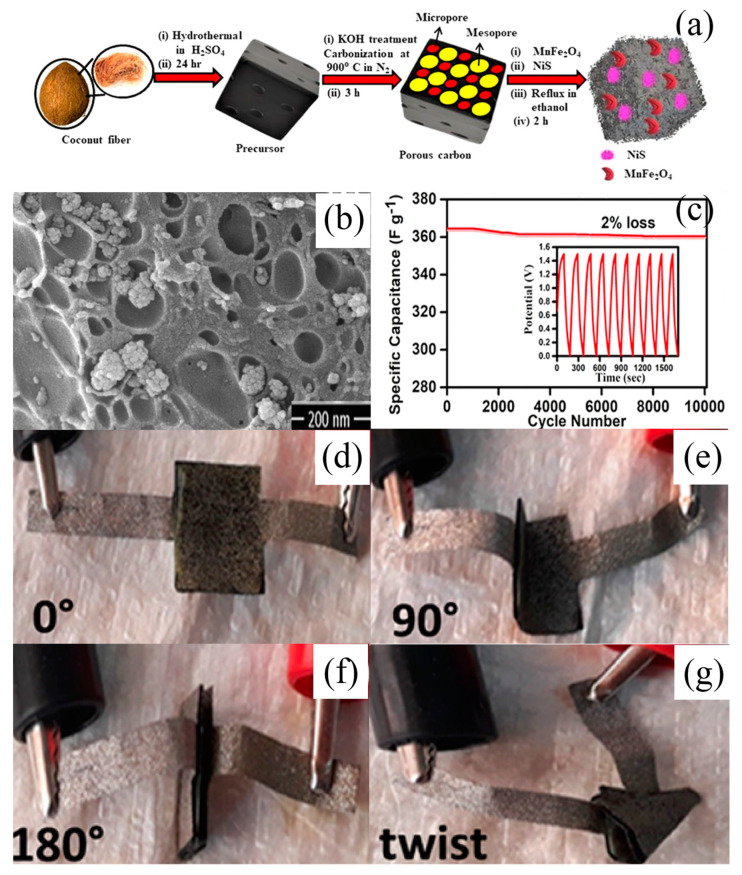
(**a**) Illustration of the synthesis of a MnFe_2_O_4_−NiS−PC nanocomposite. (**b**) Synthesized MnFe_2_O_4_−NiS−PC nanocomposite. (**c**) Cycling stability test (inset shows the GCD curves of 10 representative cycles) after 10,000 cycles. (**d**−**g**) All−solid−state ASC devices under different bending angles, demonstrating their flexible character [147].

The results of the present research show that the electrical conductivity of materials can be further improved via the synthesis of bimetallic oxides. With the increase in the material’s conductivity, the rate of electron conduction in the electrode material can be accelerated, which can help to significantly improve the magnification performance of the pseudocapacitor electrode material. In addition, the structure of bimetallic oxides often contains more Faraday redox-active sites, so the pseudocapacitance performance of bimetallic oxides is much higher than that of the corresponding monometal oxides. Mallick et al. [146] synthesized a simple N- and S-doped mesoporous carbon-supported Ni-NiWO_4_@NiS nanostructure (Ni–NiWO_4_@NiS /NS-C). A voltammetric analysis showed that the diffusion control process was the main factor affecting the capacitive properties of the materials (see Figure 14c). The hybrid supercapacitor device was composed of a synthetic Ni–NiWO_4_@NiS/NS-C material with an energy density of 43.68 Wh kg^−1^. The hybrid device had a long cycle stability (>20,000 cycles of continuous charging and discharging), and its initial capacity was increased by 34% after 2000 cycles. In addition, the researchers also prepared flexible and inflexible solid-state hybrid devices. The electrochemical analysis showed that the flexible device could still maintain its initial capacity after repeated bending at different angles. Makkar et al. [147] used biomass-derived carbon based on the easy availability and unique morphological characteristics of nanocomposites to prepare flexible all-solid-state asymmetric devices with MnFe_2_O_4_-NiS-PC as the cathode and pure porous carbon as the anode (see Figure 14d). The nanocomposites made by this team had the following advantages: (i) MnFe_2_O_4_ provided an excellent pseudocapacitance performance, and (ii) NiS was highly conductive due to its low bandgap, and the presence of various oxidation states of Ni contributed to its excellent electrochemical performance. Due to the synergy between MnFe_2_O_4_ and NiS, the device had a high power performance, which made it suitable for portable electronic devices in porous carbon networks. The device had remarkable stability at 10,000 cycles and only a 2% capacitance degradation. The electrochemical properties of the device were studied, and it was found that the device could not be damaged even under severe bending deformation (see Figure 15). 

Patil et al. [148] used a simple and cheap chemical bath deposition (CBD) method to prepare nickel sulfide films composed of highly porous nanoflames on flexible stainless steel substrates. The prepared nickel sulfide film composed of nanoflames had a good electrochemical performance in the three-electrode system, and its maximum specific capacitance was 750.6 F g^−1^ when the scanning rate was 5 mV s^−1^. Ahmad et al. [149] prepared NiS-NiCoO_4_@C composites by calcining molten salt. It was found that the CN14 composite electrode had a high specific capacitance in the three-electrode system. When the current density was 1 A g^−1^, the capacitance was 1411 F g^−1^, and the cycle life was excellent (99.5% after 2000 cycles). In addition, the ASC device (CN14//AC) achieved a high energy density of 37.2 Wh kg^−1^ at a power density of 799.9 W kg^−1^, and it maintained a good cycle performance of 89% after 5000 cycles.

The above findings show that the combination of transition metal sulfide and carbon materials as positive electrode materials provides an excellent pseudocapacitance performance, which significantly improves the conductivity and electrochemical reactivity of the electrode materials and improves the storage capacity, energy density, power density, and cycle stability of the supercapacitors. Carbon materials could be used as a skeleton to construct flexible devices. Moreover, bimetallic oxides display greater promise in this research field, and the concentration of the oxide to carbon should be studied in greater detail.

## 4. Conclusions

In this paper, we reviewed electrode materials made of nickel–sulfur, carbon, sulfide, and oxide materials and the characteristics of various electrode materials. See Table 1. Electrodes made of nickel–sulfur doped with these materials played a role in improving the theoretical specific capacity and increasing the cycle life of the capacitor. The nickel sulfide and sulfide-doped composites, as electrodes, had the advantages of good thermal stability and environmental friendliness. By designing and combining the structural characteristics of nickel–sulfur with graphene, carbon nanotubes, and conductive polymers, nanocomposite materials with a large surface area and uniform distribution of microscopic holes have been prepared by researchers, which improves the conductivity and rate performance of metal sulfide materials, and these materials are more conducive to the application in the field of supercapacitors. The composites of transition metal oxides and nickel–sulfur as electrode materials have the characteristics of a good rate performance and high cycle performance, and the working potential window of the composite materials has been much improved. However, there are still some problems that need to be solved. The following are some prospective future directions for this research topic:

(1) Studies on the cycle stability of nickel–sulfur materials without any carbon material doping have not achieved ideal results. Therefore, the cycle stability of nickel–sulfur materials can be improved by composites with carbon materials or structural designs.
(2) It is well known that the specific capacitance of oxides is relatively low. The properties of nickel–sulfur and oxide composite materials, such as nickel–sulfur and molybdenum oxide composite materials, may change when metal elements are added (e.g., doping with a small amount of nickel). In the future, this kind of material may be a valuable research direction.
(3) Elemental doping is an effective method by which to improve the electrochemical stability of battery electrodes. In particular, the codoping of two or more elements will produce synergistic effects and stabilize the bulk-phase chemistry and surface chemistry of electrode materials at the same time. In the research on carbon material doping, if the size of the carbon material is large, it is not easy to dope with nickel–sulfur materials. In addition, there are still some problems to be solved with respect to doping—for example, how to ensure a high loading rate and how to accurately control the load. Whether the nickel–sulfur material is petal shaped, granular, or flake like, it is involved in uniform distribution during the process of its dispersion to the carbon material. Further, how the morphology of the interface between nickel–sulfur materials and carbon materials changes is still a topic with research value.
(4) Research on nickel–sulfur and its composites still faces great challenges, which requires researchers to further explore easier synthesis methods considering large-scale manufacturing, cost effectiveness, and green performance.
(5) The morphology of NiS is important for its electrochemical performance. Synthesis mainly includes hydrothermal methods and sulfidation precursors. The sulfur source and reaction time should show a great influence on the phase composition and morphology. Moreover, the vulcanizing atmosphere and flow velocity are important factors. Therefore, how to precisely control these factors is also a promising direction for future studies.

## Figures and Tables

**Table 1:** Comparison of electrochemical properties of NiS based electrode materials.

Electrode Materials		Prepare Methods	Electrolyte	Specific Capacitance	Cyclic Stability	Ref.
Pure NiS nanomaterials	Porous NiS hexagonal nanoplates	Ion exchange method	2 mol L^−1^ KOH	1897 F g^−1^ (1 A g^−1^)	100% 4000 th (3 A g^−1^)	[85]
	Ultrathin NiS nanosheets	Hydrothermal	0.15 mol KOH	2587 F g^−1^ (0.2 A g^−1^)	95.8% 4000 th	[89]
	Nickel sulfide monolayer hollow spheres arrays	Ion exchange	6 M KOH	68.5 mAh g^−1^ (2 Ag^−1^)	94.8% 3000 th	[91]
	NiS hollow spheres	Electrodeposition	3 M KOH	1553 F g^−1^ (2.35 A g^−1^)	95.7% 2000 th	[94]
	Hierarchical NiS Hexagonal nanoplate	Hydrothermal	3 M KOH	606 C g^−1^ (0.5 A g^−1^)	93% 2000 th	[95]
	NiS thin film	CBD	2 M KOH	104 F g^−1^ (5 mVs^−1^)	85.3% over 3000 th	[97]
NiS/sulfide nanocomposite	Nickel sulfides	Hydrothermal	2 M KOH	194.4 mAh g^−1^ (2 A g^−1^)	89.5 mAh g^−1^ 5000 th (10 A g^−1^)	[101]
	MoS_2_/NiS	Hydrothermal	6 M KOH	1493 F g^−1^ (0.2 A g^−1^)	100% 10,000 th (0.5 A g^−1^)	[106]
	MoS_2_/NiS	Hydrothermal	6 M KOH	2225 F g^−1^ (1 A g^−1^)	94.5% 50,000 th (10 A g^−1^)	[112]
	NiO/NiS	Hydrothermal	3 M KOH	386.7 F g^−1^ (1 A g^−1^)	97.6% 3000 th (5 A g^−1^)	[114]
	NiS/SnS_2_	Cation exchange	6 mol·L^−1^ KOH	430.38 mAh g^−1^ (1.16 A g^−1^)	100% 1000 th (15 mA h^−1^)	[115]
	NiCo_2_S_4_/NiS	Hydrothermal	3 M KOH	1947.5 F g^−1^(3 mA cm^−2^)	90.3% 1000 th	[117]
	Nickel sulfides/MoS_2_	Hydrothermal	3 M KOH	676.4 F g^−1^ (1 A g^−1^)	100% 2000 th	[122]
NiS/carbon nanocomposite	A composite material combining carbon nanotubes on CoS, NiS, and nickel foam	Hydrothermal	3 M KOH	1249.88 mAh g^−1^ (1 A g^−1^)	97.17% 8000 th (1 A g^−1^)	[125]
	Electrospun carbon fibers containing NiS	Embedding NiS nanoflakes in electrospun carbon fibers	6 M KOH	1691.1 F g^−1^ (1 A g^−1^)	87.8% 5000 th (5 A g^−1^)	[126]
	Nitrogen-doped carbon-coated NiS nanoparticles	N-doped carbon-coated NiS core-shell composites	6 M KOH	1330 F g^−1^ (0.5 A g^−1^)	92.3% 3000 th (10 A g^−1^)	[127]
	NiS/reduced graphene (rGO) hybrid	Hydrothermal	2 M KOH	1745.67 F g^−1^ (1 A g^−1^)	54.2% 3000 th (10 A g^−1^)	[128]
	Graphene nanosheets (GNS)/nickel sulfide (NiS)-based material	“Dip and Dry” and Electrodeposition	6 mol L^−1^ KOH	755 F g^−1^ (0.5 A g^−1^)	88.1% 1000 th (2 A g^−1^)	[129]
	Nickel sulfide (NiS) and nitrogen-doped graphene/NiS nanocomposite	Microwave	6 M KOH	1467.8 F g^−1^ (1 A g^−1^)	86.6% 5000 th	[130]
	NiS/carbon microspheres	Hydrothermal	6 M KOH	1594 F g^−1^ (1 A g^−1^)	82.5% 4000 th	[138]
NiS/transition metal oxide nanocomposite	Nickel sulfide and reduced graphene oxide	Hydrothermal	3 M KOH	150 mAh (1 A g^−1^)	60% 3000 th (2 A g^−1^)	[141]
	NiS/Co_3_S_4_ nanosheets grown on Fe_2_O_3_ substrate	Situ solution vulcanization process	3 mol L^−1^ KOH	1213.7 F g^−1^ (1 A g^−1^)	85.2% 5000 th (5 A g^−1^)	[142]
	NiO/NiS@CNT	Microwave	6 M KOH	809.7 F g^−1^ (1 A g^−1^)	100% 20,000 th (5 A g^−1^)	[143]
	MnFe_2_O_4_−NiS−porous carbon nanocomposite	Hydrothermal	3 M KOH + 0.1 M K_4_[Fe(CN)_6_]	364 F g^−1^ (2 A g^−1^)	98% 10,000 th	[148]
	Nickel sulfide (NiS) thin film	CBD	2 M KOH	750.6 F g^−1^ (5 mV s^−1^)	85.3% over 3000 th	[149]

## Data Availability

The data used to support the findings of this study are available from the corresponding author upon request.
